# *BIFURCATE FLOWER TRUSS:* a novel locus controlling inflorescence branching in tomato contains a defective MAP kinase gene

**DOI:** 10.1093/jxb/ery076

**Published:** 2018-03-02

**Authors:** Demetryus Silva Ferreira, Zoltan Kevei, Tomasz Kurowski, Maria Esther de Noronha Fonseca, Fady Mohareb, Leonardo S Boiteux, Andrew J Thompson

**Affiliations:** 1 Cranfield Soil and Agrifood Institute, Cranfield University, Cranfield, UK; 2 National Center for Vegetable Crops Research, CNPH—Embrapa Hortaliças, Brasília-DF, Brazil

**Keywords:** *Bifurcate flower truss*, branching, genome resequencing, inflorescence architecture, low temperature, MAP kinase, *Solanum galapagense*, *Solanum lycopersicum*

## Abstract

A mutant line, *bifurcate flower truss* (*bif*), was recovered from a tomato genetics programme. Plants from the control line produced a mean of 0.16 branches per truss, whereas the value for *bif* plants was 4.1. This increase in branching was accompanied by a 3.3-fold increase in flower number and showed a significant interaction with exposure to low temperature during truss development. The control line and *bif* genomes were resequenced and the *bif* gene was mapped to a 2.01 Mbp interval on chromosome 12; all coding region polymorphisms in the interval were surveyed, and five candidate genes displaying altered protein sequences were detected. One of these genes, *SlMAPK1*, encoding a mitogen-activated protein (MAP) kinase, contained a leucine to stop codon mutation predicted to disrupt kinase function. *SlMAPK1* is an excellent candidate for *bif* because knock-out mutations of an Arabidopsis orthologue *MPK6* were reported to have increased flower number. An introgression browser was used to demonstrate that the origin of the *bif* genomic DNA at the *BIF* locus was *Solanum galapagense* and that the *SlMAPK1* null mutant is a naturally occurring allele widespread only on the Galápagos Islands. This work strongly implicates *SlMAPK1* as part of the network of genes controlling inflorescence branching in tomato.

## Teaser

The *bifurcate flower truss* (*bif*) gene, originating from *Solanum galapagense*, increases tomato inflorescence branching and flower number. A gene encoding a truncated MAP kinase is a strong candidate for *bif*.

## Introduction

Inflorescence architecture and the number of flowers produced per plant are controlled by an extensive network of genes in the Solanaceae family (Lemmon *et al.*, 2016). An increase in the number of flowers will lead to a greater fruit yield provided that reproductive growth is limited by sink strength rather than by assimilate supply (Périlleux *et al.*, 2014). Conversely, production of more flowers than the assimilate supply can sustain is a waste of resources and may negatively affect final fruit yield. Where assimilate supply is limiting, fruit number is inversely proportional to fruit size and is regulated by flower and fruit abscission in response to endogenous and environmental signals (Saglam and Yazgan, 1999). In fleshy fruit crops such as *Solanum lycopersicum* (tomato), growers also manage fruit size and uniformity by thinning and pruning (Cockshull and Ho, 1995; Max *et al.*, 2016).

The architecture of the inflorescence (referred to as a ‘truss’ in tomato), defined by peduncle length, number of branch points, and the number of flowers per unit length of peduncle, determines the potential number of fruits that can be produced. Since ripening progresses from proximal to distal truss positions (Giovannoni, 2001), fruits borne on a more branched truss, assuming the same total number of flowers, will tend to exhibit a higher degree of synchronicity in their growth and ripening (Bangerth and Ho, 1984).

Flower initiation and development have been well characterized (Lippman *et al.*, 2008), but the genetic mechanisms involved in controlling truss architecture are not fully understood. In the case of tomato, the first truss occurs after production of 6–14 leaves depending on air temperature (Atherton and Harris, 1986). After the appropriate flowering induction stimulus, the shoot apical meristem (SAM) originates the inflorescence meristem (IM), which develops floral meristems (FMs) and ultimately flowers (Lippman *et al.*, 2008; Lozano *et al.*, 2009). Truss architecture is extremely plastic and responsive to environmental factors—several studies (reviewed by Gratani, 2014) have reported variations in truss architecture in response to external signals. For example, lower temperatures increase branching and flower number (Calvert, 1957, 1959), and this is enhanced at high irradiances (Hurd and Cooper, 1967).

In monopodial plants (e.g. Arabidopsis), the SAM continues to grow and to produce new lateral growth from axillary buds during the different phases of plant development. In contrast, tomato is a sympodial plant where, after a period of growth, the SAM terminates with an inflorescence; subsequently a new vegetative cycle is initiated with the outgrowth of an axillary bud to form the new primary shoot which usually produces three new leaves (vegetative nodes) before again terminating in an inflorescence (Schmitz and Theres, 1999; Carmel-Goren *et al.*, 2003; Quinet *et al.*, 2006; Castel *et al.*, 2010; Thouet *et al.*, 2012). This pattern is repeated, forming consecutive sympodial segments which together constitute a sympodial shoot (Samach and Lotan, 2007; Kirchoff and Claßen-Bockhoff, 2013; Park *et al.*, 2014).

Although tomato and Arabidopsis have distinct growth and flowering patterns, they share a number of orthologous genes controlling inflorescence architecture. In Arabidopsis, four key genes related to meristem identity and the control of inflorescence architecture have been studied: *TERMINAL FLOWER1* (*TFL1*), *APETALA1* (*AP1*), *UNUSUAL FLOWER ORGAN* (*UFO*), and *LEAFY* (*LFY*) (Bradley *et al.*, 1997; Chandler, 2014). *TFL1* is responsible for early flowering after the development of rosette leaves; it delays the transition of IM to FM, producing a terminal flower. *AP1* is up-regulated in the FM and it negatively regulates *TFL1* and controls FM initiation. The *UFO* gene can regulate meristem identity by transforming FM back to IM (Levin and Meyerowitz, 1995), and it co-activates the *LFY* gene (Souer *et al.*, 2008) which promotes floral fate by establishing and regulating floral identity (Kardailsky *et al.*, 1999; Kobayashi *et al.*, 1999).

In tomato, six mutant genes are known to create aberrant inflorescence architectures and/or reduce flower numbers (Astola *et al.*, 2014): *falsiflora* (*Solyc03g118160*), an orthologue of *LFY*, fails to assume floral identify, remaining intermediate between vegetative and reproductive states (Allen and Sussex, 1996; Molinero-Rosales *et al.*, 1999; Lozano *et al.*, 2009); *anantha* (*Solyc02g081670*), an orthologue of *UFO* (Souer *et al.*, 2008), causes the IM to propagate indefinitely, producing large inflorescences with immature flowers resembling the arrested inflorescences of cauliflower curd (Allen and Sussex, 1996); *jointless* (*Solyc11g010570*) produces an FM, but after 3–4 flowers the IM is converted to a vegetative meristem (VM) (Szymkowiak and Irish, 1999; Mao *et al.*, 2000); *blind* (*Solyc11g069030*) affects meristematic development during the vegetative stage and reduces the number of inflorescences and flowers (Schmitz *et al.*, 2002); *uniflora* (*Solyc09g005070*), orthologous to the rice *LAX PANICLE* and maize *BARREN STALK1* genes, produces only a single flower due to the inability to control the transition between IM and FM (Mero and Honma, 1982; Dielen *et al.*, 2004; Quinet *et al.*, 2011); *terminating flower* (*Solyc09g090180*) shows early inflorescences with a single, abnormal flower (MacAlister *et al.*, 2012), and the wild type allele up-regulates vegetative growth by suppressing *FALSIFLORA* expression (Périlleux *et al.*, 2014); it encodes a transcription factor that interacts with BTB/POZ transcriptional regulators (Xu *et al.*, 2016).

In addition, there are three mutations that increase branching and flower number on an otherwise normal inflorescence: first, *bifurcate inflorescence* (*bi*) was reported to cause the inflorescence to branch at least once and to reside on chromosome 5 (Burdick and Mertens, 1955). However, the authors are not aware of any further characterization of *bi*. The second mutant gene, *compound inflorescence* (*s*), increases the number of flowers by increasing the number of peduncle branch points. The *S* gene (*Solyc02g077390*) encodes a transcription factor related to WUSCHEL HOMEOBOX located on the long arm of chromosome 2 (Lippman *et al.*, 2008). The third gene known to increase inflorescence branching is *jointless2* (*j2*) located in the centromeric region of chromosome 12; its primary phenotype is the lack of a pedicel abscission zone, but it is known to be associated with a bifurcate truss (Reynard, 1961), and it has been suggested that this was due to linkage drag (Roldan *et al.*, 2017) because knocking out *j2* alone by gene editing did not affect inflorescence branching. However, recently it was discovered that mutations in the two redundant MADS box genes *j2* (*Solyc12g038510*) and the unlinked *enhancer-of-j2* (*ej2*; *Solyc03g114840*) caused increased inflorescence branching by epistatic interaction (Soyk *et al.*, 2017).

In this study we identify a tomato mutation at a novel locus *BIFURCATE FLOWER TRUSS* (*BIF*) which produces a highly branched inflorescence similar to that produced by *s*; we describe the phenotype of *bif* and identify one strong candidate gene on chromosome 12 by fine genetic mapping and bioinformatic analysis.

## Materials and methods

### Plant material

Seeds of the inbred lines LAM183 and *bif* were obtained from our co-operative tomato genetics programme. After growth and self-pollination under glasshouse conditions in the UK (Cranfield University), plants of *bif* and LAM183 that clearly showed high or low branching, respectively, were selected and self-pollinated, and regrown to confirm that the contrasting branching phenotypes were inherited in all progeny prior to phenotyping. These stable inbred lines were also used to produce LAM183 × *bif* F_1_ and F_2_ seeds for genetic mapping (Supplementary Fig. S1 at *JXB* online).

### Plant growth

Seeds were sown in 9 cm Petri dishes containing two layers of Whatman No. 1 filter paper soaked with 3 ml of tap water and placed in the dark at 25 °C for 3 d. Chitted seeds were sown into 3 litre pots, 15 cm diameter×18 cm height, in a glasshouse in Sinclair multipurpose compost (LBS Horticulture Ltd, Colne, UK). Glasshouse day/night temperature set points, unless otherwise specified, were 20/18 °C, with supplementary light provided by high-pressure sodium lamps. Pots were irrigated according to demand, and were fed twice a week with Hoagland solution at half concentration before flowering and full concentration after flowering.

### Cold experiment

Sixty chitted seeds from each inbred line (*bif* accession WSS3666; LAM183 accession WSS3674) were potted in the glasshouse in three randomized blocks (*n*=20 plants per genotype per block) and left for 1 week in the glasshouse (set point 23/23 °C day/night) for initial establishment. Groups of 24 plants (6 replicates×2 genotypes×2 temperatures) were transferred at five different stages of development to two growth cabinets set to provide either a cold treatment (15 °C) or a control treatment (23 °C), as shown in Supplementary Fig. S2. There was a 14/10 h day/night regime with 208 µmol m^−2^ s^−1^ photosynthetic photon flux from cool white fluorescent bulbs at 80% relative humidity. After 4 d at the differential temperature, the plants were transferred back to the glasshouse, maintaining the randomized block design. After the last transfer (i.e. week 6), all plants were grown in the glasshouse at a minimum temperature of 23 °C until the third truss was formed and the first two trusses were mature enough to be scored for branching and flower number.

### Next-generation sequencing genomic data generation and variant calling

Genomic DNA from LAM183 and *bif* plants was extracted using the DNeasy plant mini kit (Qiagen, Manchester, UK). One lane of a HiSeq 2500 (Illumina, Saffron Walden, UK) was used to sequence each genome, using 126 bp paired-end reads. Quality control was performed by FastQC (Schmieder and Edwards, 2011) to ensure a QC average of >33. The reads were mapped to the tomato Heinz 1706 reference genome SL2.50 (Sato *et al.*, 2012): first, the reads were aligned by the Burrows–Wheeler aligner (BWA, version 0.7.4), using default specifications; secondly, the aligned reads were compressed into a binary (bam) format (Picard tools) and then sorted and indexed by Samtools (version 0.1.19); thirdly, the GATK package (Genome Analysis Tool Kit, Broad Institute, Cambridge, USA, version 3.3.0) was used to realign the insertions and deletions (InDels) and for variant calling (HaplotypeCaller, using default settings). This pipeline produced variant call format (VCF) files which were annotated by SnpEff (version 4.0) using ITAG2.40, associated with genome reference version SL2.50) (http://solgenomics.net/, last accessed 12 March, 2018; Fernandez-Pozo *et al.*, 2015). Finally, the variants were filtered using GATK’s variant filtration tool (Quality Depth <2, Fisher Strand >60, Mapping Quality <40, Haplotype Score >13, and Mapping Quality RankSum <12.5) (Kevei *et al.*, 2015). Unique variants were filtered using a custom BASH script which excluded polymorphisms shared between the data sets, similar to the mechanism of bedtools (Quinlan and Hall, 2010). The NCBI BioProject accession for the raw reads and VCF files is PRJNA378916. The VCF files were uploaded to the GenoVerse Genome Browser (Bragin, 2012): http://elvis.misc.cranfield.ac.uk/GenoverseBIF, last accessed 12 March, 2018. Additional predictions of the effects of amino acid substitutions on protein function were performed using the PROVEAN tool (Choi *et al.*, 2012): http://provean.jcvi.org/index.php, last accessed 12 March, 2018.

### Single nucleotide polymorphism (SNP) genotyping

Using the Genoverse genome browser, 48 SolCap markers (Sim *et al.*, 2012) observed to be polymorphic between LAM183 and *bif* were manually selected to provide two on each chromosome arm outside of the heterochromatin. DNA extraction from leaf tissue of individual plants of the LAM183×*bif* F_2_ population (*n*=96) and Kompetitive Allele Specific PCR (KASP) genotyping of the 48 SolCap markers (Supplementary Table S1) was performed by LGC (Teddington, UK). For other specifically designed KASP marker assays, genomic DNA was extracted using a protocol based on Chelex-100 (Bio-Rad, Hemel Hempstead, UK) with modifications (Walsh *et al.*, 1991; Supplementary Data S1).

### Genotyping by PCR-based markers

Reactions were performed using 1 µl of purified genomic DNA (~50 ng) in a 10 µl reaction volume containing 1× KASP master mix buffer and 1× KASP-specific primer mix (LGC). Using a CFX96 real-time PCR machine (Bio-Rad, Hemel Hempstead, UK), thermal cycling was initiated at 94 °C for 15 min, followed by nine cycles of 94 °C for 20 s, 61–55 °C for 1 min (0.6 °C drop per cycle), and then 25 cycles of 94 °C for 20 s, 55 °C for 1 min. The temperature was decreased to 37 °C for 1 min for the final step of fluorescent plate reading. KASP assays used fluorophores FAM and HEX for distinguishing genotypes; results were analysed in the ‘Allelic Discrimination’ feature of CFX manager (BioRad). All KASP assay primers were developed by LGC based on sequence data provided to them (Supplementary Table S2).

### Statistical analysis

Sample SD, SE, and ANOVA were calculated using SigmaPlot (Systat Software Inc., Hounslow, UK). For ANOVA, significant differences were claimed if *P* was <0.05 in a Tukey and Dunn’s post-hoc test. Data were transformed prior to ANOVA to ensure the validity of the normality assumption: for flower number, a log(*x*) transformation was used; for branch point number (containing zero values), a log(*x*+1) transformation was used. Data were back-transformed prior to plotting.

### Similarity map analysis

iBrowser script (Aflitos *et al.*, 2015) was used to extract homozygous SNPs from VCF files and to generate FASTA sequences, distance matrices, and Newick trees for segments listed in general feature format (GFF) files. The GFF files were generated using a custom BASH script which split the interval of interest into evenly sized segments. iBrowser webserver scripts generated the final clustering and introgression plots. SNP data for this analysis originated from resequencing of tomato cultivar and wild species accessions (Aflitos *et al.*, 2014; Lin *et al.*, 2014).

## Results

### The *bif* phenotype: initial characterization and pedigree

Multiple tomato germplasm lines and hybrids were crossed in a single seed descent programme with the aim of obtaining small elongated fruits (‘miniplum’ or ‘grape’ type) combining high yield and high Brix values. One inbred line was selected due to its high number of branch points and flowers, and it was named *bif* due to the increased truss branching. LAM183 was an alternative inbred line developed from the same breeding programme with similar fruit morphology, high Brix value, and general growth habit, but lacking the increase in truss branching. LAM183 and *bif* lines are therefore phenotypically similar with the exception of truss branching, but the precise pedigree and genetic differences between them were unknown at the initiation of this study.

### Truss development and characterization in the contrasting inbred lines

**Fig. 1.**
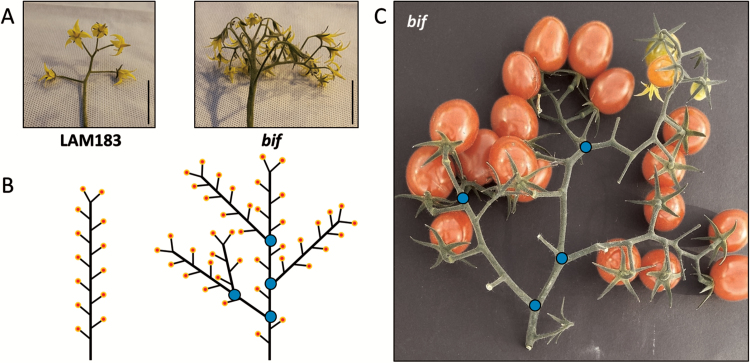
Truss architecture in LAM183 and *bif*. (A) Images of representative flowering first trusses, 30 d after germination. (B) Schematic diagram illustrating the mean number of flowers (yellow and red circles) and branch points in the first truss (means taken from Table 1). (C) Image of an example *bif* truss at the fruiting stage. In (A), the black scale bars represent 5 cm. In (B) and (C), blue circles indicate the position of branch points. In (A) and (B), the identity of the LAM183 and *bif* images is indicated.

The most obvious phenotypic difference between *bif* and LAM183 was the higher number of flowers produced on *bif* trusses (Fig. 1). Considering the mean of the first two trusses, *bif* produced 39.8 ± 1.6 flowers per truss, which was 3.3-fold higher than the 12.0 ± 0.3 flowers per truss exhibited by LAM183 (Table 1). The number of truss branch points was also affected—*bif* trusses showed a mean of 4.1 ± 1.8 branch points per truss compared with 0.16 ± 0.37 in LAM183, representing a 25.6-fold difference. Both trait values were significantly higher in *bif* (Table 1). Other minor, but statistically significant, phenotype observations were that *bif* plants were taller with a more vigorous early root development, and had larger seeds (Table 1).

**Table 1.** Phenotypic characterization of LAM183 and *bif* parental lines

Trait	LAM183		*bif*	
	First truss	Second truss	First truss	Second truss
Flowers per truss	12.85 ± 0.42 a	11.08 ± 0.38 b	41.81 ± 2.77 c	37.75 ± 1.66 c
Branch points per truss	0.12 ± 0.04 a	0.20 ± 0.05 a	4.37 ± 0.31 b	3.81 ± 0.16 b
Leaves before the first truss	6.66 ± 0.21 a		6.86 ± 0.21 a	
Plant height at 61 d (cm)	87.7 ± 3.7 a		119.1 ± 2.7 b	
Tap root length at 13 DAG (cm)	5.4 ± 0.1 a		10.4 ± 0.2 b	
Seeds per fruit	46.1 ± 1.1 a		45.9 ± 1.4 a	
Seed area (mm^2^ per seed)	5.8 ± 0.04 a		7.6 ± 0.05 b	

Significant differences (Students *t*-test) are represented by different letters (*P*<0.05). Errors are the SE; different population sizes were used for each trait: *n*=15 (plant height), *n*=20 (seeds per fruits); *n*=30 (tap root length), and *n*=48 (number of flowers and branch points/truss). DAG, days after germination.

LAM183 plants exhibited branching in some trusses, and occasionally unbranched trusses were found in the first truss of *bif* plants, although scoring of plants was unambiguous when looking at multiple trusses in older plants. Thus, truss position and a genotype×environment interaction were apparently affecting the penetrance of this trait.

### Environmental interactions—cold effect

**Fig. 2.**
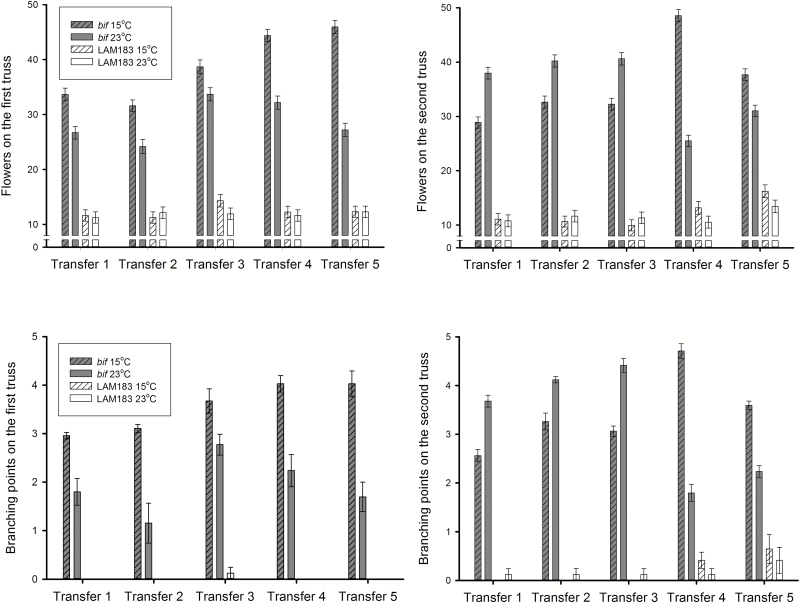
Effects of low temperature on truss branching and flower number. The numbers of flowers and branch points were recorded at 47 d after germination in a population of 60 plants per genotype. Error bars represent the SE (*n*=6). ANOVA results are summarized in Table 2.

The effects of low temperature treatments on tomato truss architecture are well established (Calvert, 1957, 1959). When LAM183 was grown in Brasília, truss branching was very rarely observed, whereas in the lower temperatures typical of the UK there appeared to be a more frequent incidence of branching (mean of first and second trusses=0.16 branches per truss; Table 1). Therefore, an experiment was conducted to test whether low temperature could induce truss branching, and if there was an interaction between genotype and temperature. LAM183 and *bif* plants were transferred from a glasshouse at 23 °C to growth cabinets either at 15 °C (cold) or at 23 °C (control) for 4 d periods at weekly intervals over 5 weeks during initiation and development of trusses (Supplementary Fig. S2), and then the subsequent truss development was recorded (Fig. 2). There were more flowers and branch points in *bif* than in LAM183 (*P*<0.001; Table 2) at both the first and second trusses, as expected.

**Table 2.** Cold transfer experiment: summary of ANOVA

	Flower number				Branch points			
	First truss		Second truss		First truss		Second truss	
	*P*	LSD	*P*	LSD	*P*	LSD	*P*	LSD
Genotype (G)	**<0.001**	0.112	**<0.001**	0.089	**<0.001**	0.143	**<0.001**	0.118
Treatment (T)	**0.004**	0.112	0.446	0.089	**0.001**	0.143	0.549	0.118
Transfer point (TP)	0.126	0.177	0.180	0.140	0.435	0.226	0.610	0.186
G×T	**0.025**	0.158	0.838	0.125	**0.004**	0.202	0.635	0.167
G×TP	0.574	0.251	0.69	0.198	0.646	0.320	**0.015**	0.263
T×TP	0.909	0.251	**<0.001**	0.198	0.929	0.320	**<0.001**	0.263
G×T×TP	0.611	0.354	0.130	0.280	0.749	0.453	0.355	0.372

*P*-values <0.05 are highlighted in bold. Least significant differences (LSDs) are given at the 5% level. Treatment was a transfer to 15 °C or 23 °C for 4 d at five different transfer points (see Supplementary Fig. S2). Means are given in Fig. 2.

The cold treatment significantly increased the number of flowers produced on the first truss of *bif* (cold=38.86 ± 2.83; control=28.80 ± 1.78) regardless of when the plants were exposed to the lower temperature (Table 2; Fig. 2). The same effect was not seen in the first truss of LAM183 (cold=12.36 ± 0.52; control=11.85 ± 0.18; Fig. 2), and the response of the two genotypes to cold was significantly different (*P*=0.025 for the genotype×treatment interaction; Table 2).

On the second truss, there was a significant interaction between treatment and transfer point (*P*<0.001; Table 2) because the cold treatment had opposite effects depending on whether the transfer was early or late in truss development: the cold treatment significantly reduced the number of flowers in *bif* up to and including the third transfer (means of the first three transfers: cold=31.26 ± 1.89; control=39.61 ± 0.82; Fig. 2); after this threshold, the exposure to lower temperature increased the number of flowers produced (means of the last two transfers: cold=43.13 ± 5.43; control=28.27 ± 2.75; Fig. 2). This effect of the later transfers on the second truss was similar to that observed for the first truss at all five transfer points.

In contrast to the first truss, although the impact of cold treatment on flower number in the second truss was smaller in LAM183 compared with the *bif* line, there was no statistical evidence of a different pattern of behaviour between genotypes because the genotype×treatment (*P*=0.838) and genotype×treatment×transfer point (*P*=0.130) interactions were not significant (Table 2).

As expected, the number of branch points followed a similar pattern to that exhibited by the number of flowers (Fig. 2). On the first truss, the *bif* plants showed a significant increase in branch points in the cold treatment (cold=3.55 ± 0.22; control=1.93 ± 0.27), compared with a non-significant difference in LAM183 (cold=0.02 ± 0.02; control=0), and there was a significant genotype×treatment interaction (*P*<0.004; Table 2), but no interaction with transfer point, similar to the observation for flower number for the first truss.

For the second truss, *bif* was less branched when exposed to lower temperatures up to and including the third transfer (means of the first three transfers: cold=2.96 ± 0.22; control=4.07 ± 0.21), and the effect was inverted by the fourth and fifth transfers (cold=4.14 ± 0.55; control=2.01 ± 0.23), resulting in a highly significant treatment×transfer point interaction (*P*<0.001; Table 2). However, as observed for flower numbers, there was no statistical evidence that the branching response of the two genotypes to cold was different in the second truss (Table 2; Fig. 2).

In summary, a significant genotype×temperature interaction was observed for the first truss where *bif* responded more strongly than LAM183 to low temperature by producing a proportionally greater increase in flower numbers and branch points. However, this difference was not significant in the second truss.

### Initial genetic analysis of the *BIF* locus

**Fig. 3.**
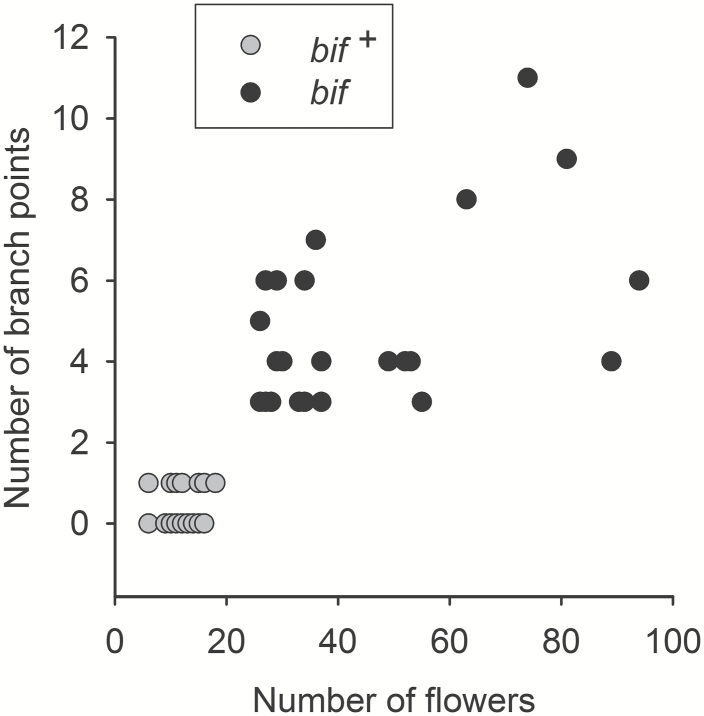
Phenotype scores for *bif* in a LAM183×*bif* F_2_ population. Phenotype was scored 52 d after germination in a population of 96 plants in which 71 were scored as wild type (*bif*^*+*^) and 25 as *bif.* Plants with 0 or 1 branch points and ≤18 flowers on the first truss were scored as *bif*^*+*^. All 96 data points are plotted, but some are superimposed within each class as they have identical values.

A LAM183×*bif* F_2_ population was grown and 96 plants were scored by observing the first truss. A plot of flower number versus branch point number showed two clear clusters of plants (Fig. 3), allowing plants to be scored as wild type (*bif*^*+*^) if they had 0 or 1 branch points and ≤18 flowers on the first truss. Conversely, plants were scored as *bif* if they had three or more branch points and ≥26 flowers. Phenotype scores were confirmed in some plants by observing the same patterns in multiple trusses in later development. It was notable that the variation within the *bif* class was considerably greater than for the *bif*^*+*^ class (Fig. 3). Twenty-five plants were scored as *bif* and 71 plants were scored as wild type (*bif*^*+*^). A χ^2^ test indicated no significant deviation from a 3:1 segregation ratio (*P*=0.814), and F_1_ plants were phenotypically similar to LAM183; therefore, *bif* behaves as a single recessive gene. In order to map the *BIF* locus genetically, the LAM183 and *bif* lines were resequenced to obtain polymorphic markers.

### Resequencing of *bif* and *LAM183* inbred lines

Illumina sequencing of genomic DNA resulted in 148 million paired-end 126 bp reads for LAM183 and 138 million reads for *bif*. The raw reads were mapped to the tomato reference genome and gave 33- and 34-fold coverage for LAM183 and *bif*, respectively. Both inbred parental lines came from single seed descent from a population with a relatively large genetic base, so a high degree of polymorphism was expected at multiple loci. After filtration to ensure that only high-quality SNPs that were polymorphic between *bif* and LAM183 were included, plots were created with 625 887 unique *bif* and 479 247 unique LAM183 SNPs (Supplementary Fig. S3), and with 77 049 unique *bif* and 81 894 unique LAM183 InDels (Supplementary Fig. S4). In total, there were 1 264 077 polymorphisms between the two genomes, and the distribution pattern of SNPs and InDels was similar.

These plots enabled a genome-scale evaluation of the genetic differences between the *bif* and LAM183 sibling lines used as parents in the genetic mapping of the *BIF* locus. Chromosomes 2, 3, and 10 showed very little polymorphism between the two lines, whereas chromosomes 5, 11, and 12 were very different over the majority of the chromosome lengths. On chromosome 12, the *bif* line diverged more than LAM183 from the Heinz 1706 reference genome, whereas on chromosome 11 the opposite was true. On chromosome 5, there was extensive polymorphism between *bif* and LAM183, and both lines were similarly divergent to Heinz 1706. On chromosomes 1, 4, 6, 7, 8, and 9, there were localized regions in which a high degree of polymorphism between *bif* and LAM183 was observed, notably with a large peak at ~30–42 Mbp on chromosome 6 where only LAM183 was highly divergent to Heinz 1706 (Supplementary Fig. S3, S4). Both *bif* and LAM183 possess the wild-type allele of *compound inflorescence* (*s*^*+*^), thus excluding this gene as the cause of truss branching in *bif*.

**Fig. 4.**
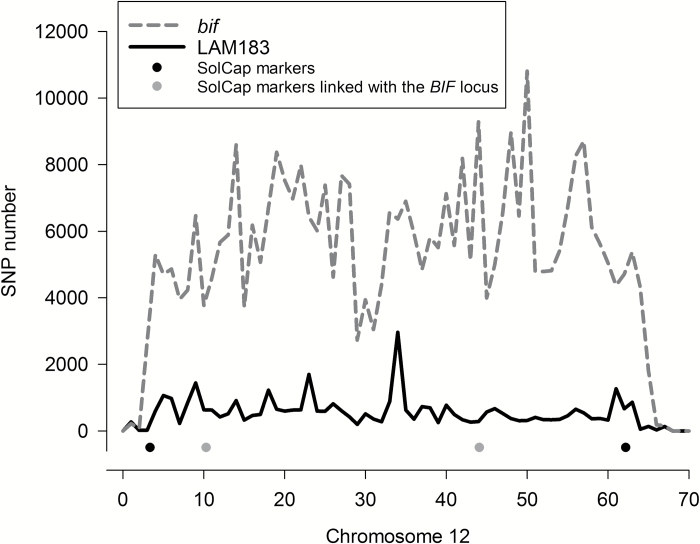
SNP density and discovery of SNP markers linked to *bif* on chromosome 12. SNPs relative to the reference genome (*Solanum lycopersicum* cv. Heinz 1706 SL2.50) are plotted using 1 Mbp bins. Only unique SNPs that are polymorphic between *bif* and LAM183 are shown. The four dots represent the SolCap markers used for initial mapping. Markers indicated by the grey dots are those linked to *bif*, namely DSF46 at 10.6 Mbp and DSF47 at 44.1 Mbp (see Supplementary Table S3). SNP and InDel plots for all chromosomes are provided in Supplementary Figs S3 and S4, respectively.

The variant calling was used to identify two SolCap SNP markers on each chromosome arm that were polymorphic between *bif* and LAM183 (Supplementary Table S1). An F_2_ population of 96 plants was genotyped with these markers, and linkage analyses (Supplementary Table S3) showed that the *bif* phenotype was linked with two markers on chromosome 12 (DSF46 and DSF47) which closely flanked each end of the central heterochromatic region (Fig. 4).

### Higher resolution gene mapping

The population of 96 F_2_ plants was genotyped with additional markers (DSF50–DSF60; Supplementary Table S2) to narrow the mapping interval from a length of 59.05 Mbp (DSF45: 3 036 369 bp–DSF48: 62 088 020 bp) to a length of 44.08 Mbp (DSF53: 7 479 839 bp–DSF56: 51 569 050 bp). DSF53 and DSF56 were then used to screen an F_2_ population of 6000 plants: 600 recombinants were recovered and phenotyped. A new batch of seven markers (DSF61–DSF67) was used to genotype the recombinants, and the mapping interval was reduced to 3.68 Mbp (DSF53: 7 479 839 bp–DSF61: 11 159 684 bp). Nineteen of the recombinants were genotyped with additional markers (DSF68–DSF72) and this defined a 2.01 Mbp region (Table 3) containing the *BIF* gene (DSF68: 8 566 567–DSF71: 10 579 861). This region encompasses 53 gene models according to the ITAG 2.40 annotation.

**Table 3.**
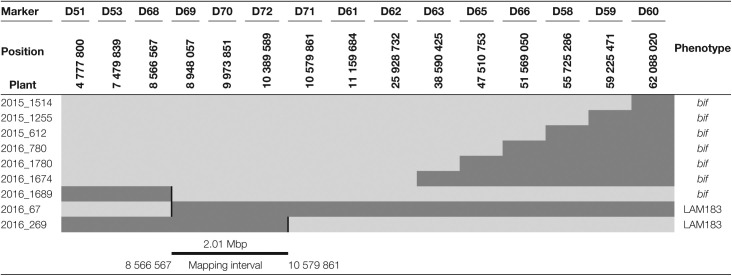
Genotyping of recombinants for fine mapping of *bif*

Results were summarized using representative recombinants. Genotype scores are coded with greyscale: light grey, homozygous (*bif.bif*); dark grey, heterozygous (*bif*.*bif*^*+*^). Marker names are symbolized by the first letter (e.g. DSF 51=D51) and positions are represented in base pairs on chromosome 12 (reference SL2.50).

### Candidate gene analyses

Of the 53 annotated genes, 22 are unlikely to be functional: four are transposons and 18 are apparently artefactual genes with no expression recorded in the TomExpress RNAseq database (Zouine *et al.*, 2017) (http://gbf.toulouse.inra.fr/tomexpress/, last accessed 12 March, 2018) or were annotated as consisting of an incomplete protein structure often with a single short exon (ITAG 2.40; http://solgenomics.net/, last accessed 12 March, 2018). Another 15 genes have coding regions that only contain silent synonymous amino acid changes between *bif* and LAM183, and a further 11 contain conservative missense polymorphisms that are less likely to cause protein function changes than non-conservative differences.

Of the remaining five candidate genes, four have non-conservative polymorphisms (*Solyc12g019130*, *Solyc12g019140*, *Solyc12g019200*, and *Solyc12g019320*; Table 4). However, the PROVEAN tool predicted that only two of these genes contained amino acid changes with a deleterious effect (Table 4): (i) *Solyc12g019130* encoding a polygalacturonase predominantly expressed in the roots (Supplementary Fig. S5) and (ii) *Solyc12g019320* encoding a putative MATE transporter, a class of genes involved in transmembrane transport of metabolites, which is expressed in all tissues except mature fruit (Supplementary Fig. S5). Neither class of gene has a reported functional role in inflorescence branching.

**Table 4.** Candidate genes in the *BIF* mapping interval containing non-conservative amino acid substitutions or nonsense mutations

Gene identity	Nucleotide position (SL2.50)	Protein annotation	Non- conservative amino acid substitution	PROVEAN prediction (score)	LA0528	LA1044	LA0483	LA1401
*Solyc12g019130*	9244294–9245985	Polygalacturonase	T381A	Neutral (0.006)	✓	✓	✓	✓
			G194R	Deleterious (–8.000)	×	✓	×	×
*Solyc12g019140*	9293132–9295123	Polygalacturonase	D206G	Neutral (–0.116)	✓	✓	✓	✓
*Solyc12g019200*	9547294–9548500	RING-finger protein-like	Q29P	Neutral (–0.867)	✓	✓	✓	✓
			G35C	Neutral (–2.586)	×	✓	✓	✓
*Solyc12g019320*	9971385–9976838	MATE transporter	I459T	Deleterious (–2.926)	×	✓	✓	✓
*Solyc12g019460*	10385358–10395971	MAP kinase 1 (mpk1=SlMAPK1)	L291*	Nonsense (n/a)	×	✓	✓	✓

For each gene, the non-conservative amino acid substitutions from LAM183 to BIF are given. Outputs from the PROVEAN tool are given as a predicted effect on protein function and the associated score. Variants with a score equal to or below –2.6 are considered ‘deleterious’ and variants with a score above –2.6 are considered ‘neutral’; PROVEAN was not able to generate a score from the protein with the early stop codon*, hence it is not applicable (n/a). The co-occurrence of a SNP in *bif* and any of the four *S. galapagense* accessions LA0528, LA1044, LA0483, or LA1401 is indicated by a tick. Expression patterns of these genes are given in Supplementary Fig. S5.

The final gene in the interval, *Solyc12g019460*, annotated as a mitogen-activated protein (MAP) kinase contains the highest impact polymorphism: an SNP that creates a new stop codon in the fifth exon (Table 4). This gene was named *SlMAPK1* in a systematic survey of tomato MAP kinases (Kong *et al.*, 2012). The predicted protein sequence of the LAM183 allele of this gene is 396 amino acids, but the *bif* allele lacks 106 amino acids at the C-terminus due to the conversion of a leucine at position 291 to a stop codon (L291*) (Supplementary Fig. S6). This truncation also removes 58 amino acids from the C-terminal end of the protein kinase domain (pfam00069) and is highly likely to result in a null allele.

Expression of *SlMAPK1* was recorded at similar levels in all tissues in the TomExpress RNAseq database (349 RNA samples from 222 conditions), but with higher expression in pollen and roots. In a study targeted to all tomato MAPK genes, expression of *SlMAPK1* was shown to occur at similar levels in all stages of flower development from 2 mm buds to 2 d after opening, but was more highly expressed in the stamen than in petals or pistils (Kong *et al.* 2012).

### Germplasm origin of the genomic region encompassing the *BIF* locus

Sequence similarities across the whole of chromosome 12 between *bif*, LAM183, and 87 accessions, including 55 *Solanum lycopersicum* accessions and 30 accessions from 11 other wild species (Aflitos *et al.*, 2014), were visualized using an introgression browser (Aflitos *et al.*, 2015) at a resolution of 50 kbp (Supplementary Fig. S7). In order to improve the contrast between more similar accessions, the analysis was repeated with 65 accessions after excluding those that were most distantly related: *bif*, LAM183, Heinz 1706, three *S. pimpinellifolium* accessions, four *S. galapagense* accessions, and the 55 *S. lycopersicum* accessions were included (Supplementary Fig. S8). All additional resequencing data were from Aflitos *et al.* (2014), apart from *S. galapagense* LA0528 which was from Lin *et al.* (2014). The results are consistent with the *bif* line containing an introgression from *S. galapagense* spanning from 2.5 Mbp to 63.5 Mbp, with the remaining distal parts of the chromosome arms being more similar to *S. lycopersicum.*

**Fig. 5.**
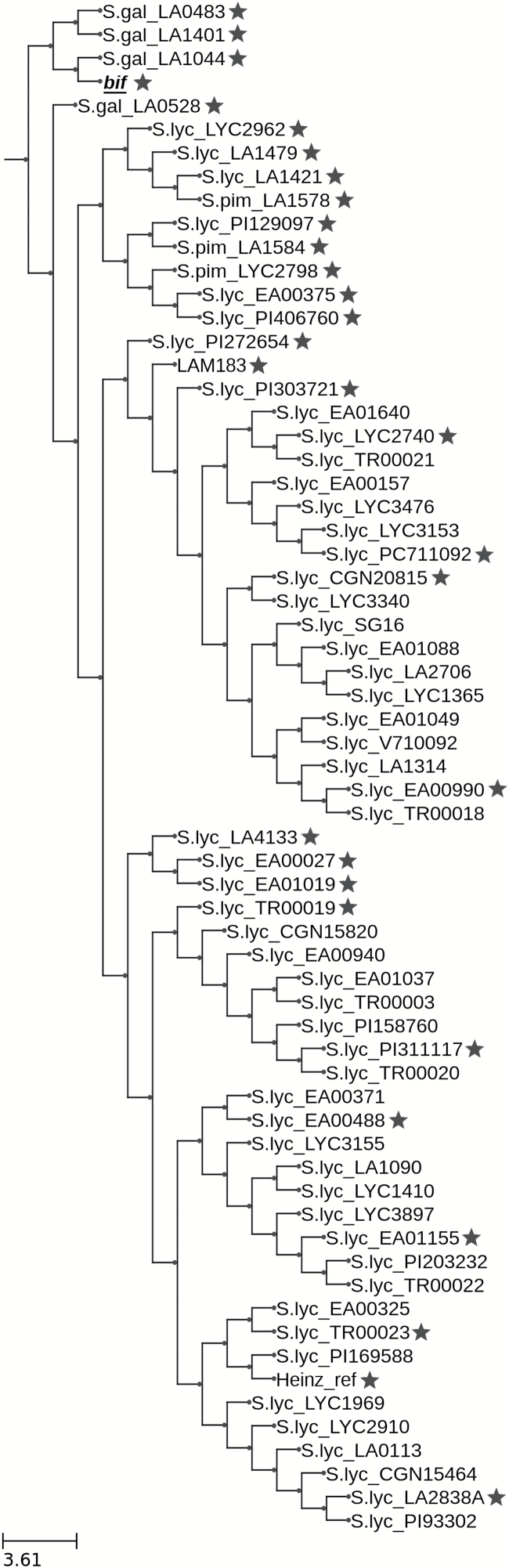
Similarity tree based on the SNPs in the 2.01 Mbp *BIF* mapping interval on chromosome 12. The tree includes the subset of the 84 resequenced accessions (Aflitos *et al.*, 2014) that excludes 23 of the wild species accessions that were most distant from *bif*. It also includes S.gal_LA0528 from Lin *et al*. (2015). Black stars show the representative lines selected to be part of the heat map of the mapping interval in Fig. 6. S. lyc, *Solanum lycopersicum*; S. gal, *Solanum galapagense*; S. pim, *Solanum pimpinellifolium*; ref, *S. lycopersicum* c.v. Heinz 1706.

**Fig. 6.**
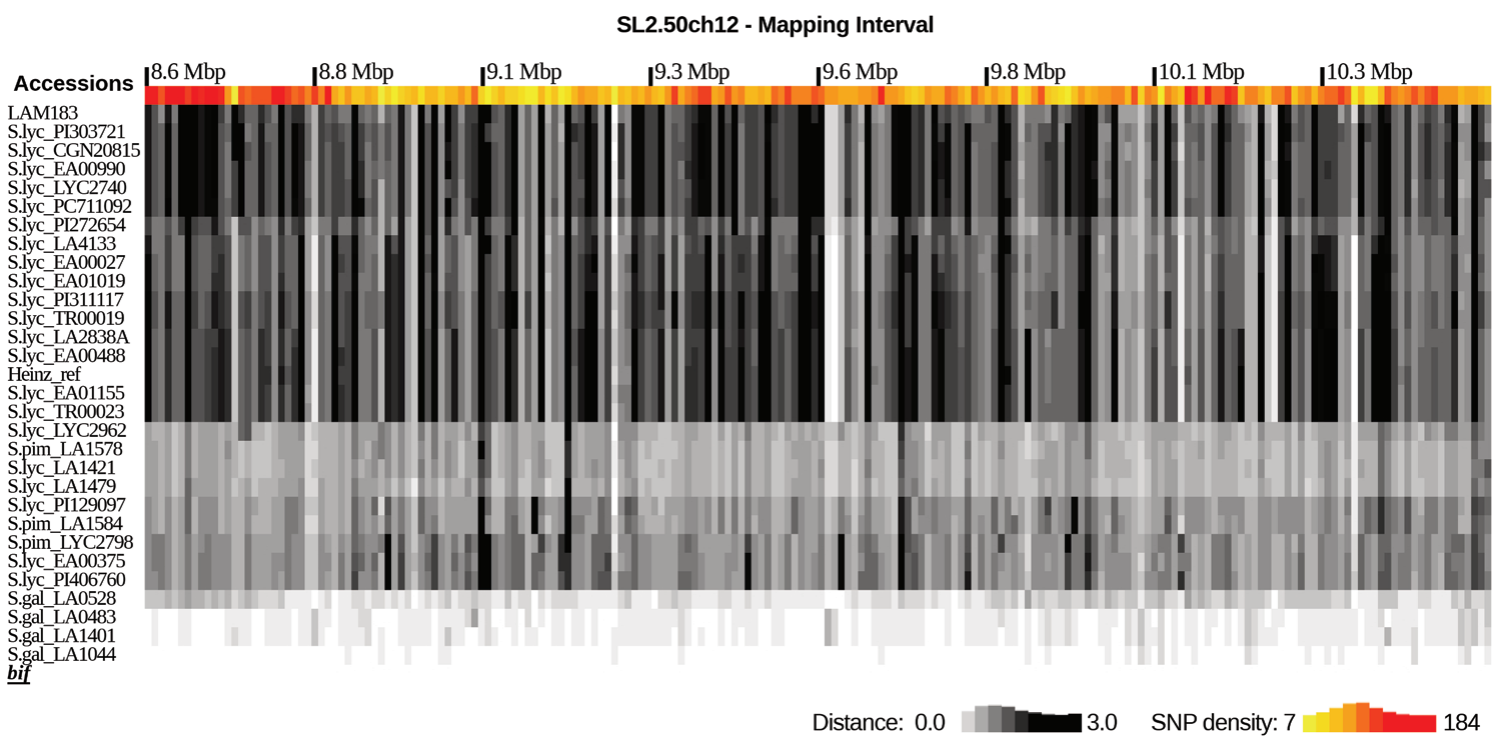
SNP heat map of the *BIF* 2.01 Mbp mapping interval on chromosome 12. Selected lines, as indicated by asterisks in Fig. 5, are shown for brevity. A more extensive analysis with 64 accessions is shown in Supplementary Fig. S9. The grey scale represents the number of SNPs in comparison with *bif*, with a larger number of SNPs giving a darker shade. The colour scale represents the SNP density across all lines. Each bin represents 10 kb. *S. lyc*, *Solanum lycopersicum*; *S. gal*, *S. galapagense*; *S. pim*, *S. pimpinellifolium*; Heinz_ref, *S. lycopersicum* c.v. Heinz 1706.

For the 65 accessions used in the whole chromosome comparison (Supplementary Fig. S8), a similarity tree (Fig. 5) and similarity heat map (Supplementary Fig. S9) were created focusing only on the mapping interval from 8.6 Mbp to 10.6 Mbp and at a higher resolution of 10 kbp. For clarity and brevity, the heat map is also displayed with *bif* compared with 31 selected accessions (Fig. 6). The data show that the *BIF* mapping interval is very similar to that of *S. galapagense* accessions LA1044, LA1401, and LA0483, with LA1044 being the most similar. These three accessions contain the same large-effect L291* SNP on the fifth exon of *Solyc12g019460* as observed in *bif.* No other tomato accessions have this allele based on resequencing data of 444 accessions (Aflitos *et al.*, 2014; Lin *et al.*, 2014). The *S. galapagense* accession LA0528 is more distant from *bif*, falling in a different clade (Fig. 5), and lacks the L291* SNP (Table 4).

## Discussion

Considerable recent advances have contributed to our understanding of the regulation of truss branching in tomato, but the molecular control of this process remains incompletely understood. In this study, a novel locus controlling truss architecture, *BIF*, was identified and characterized.

### The origin of *bif*

The phylogenetic tree of the 2.01 Mbp *BIF* mapping interval shows that the *bif* allele and surrounding genomic DNA sequence is very closely related to three *S. galapagense* accessions, with LA1044 being the closest (Fig. 5). All three accessions contain the L291* large-effect SNP, whereas it is not detected in 441 other accessions of various tomato wild species and cultivars; this strongly suggests an origin of this DNA from *S. galapagense*.

Although the inflorescence architecture of these three *S. galapagense* accessions has not been reported, *S. galapagense* LA0317 presented only one branch per inflorescence (character state=4) (Peralta and Spooner, 2005), and the collection record of LA1044 in the Tomato Genetics Resource Centre database (tgrc.ucdavis.edu) noted that it was probably part of the same population as LA0317. Also, *S. galapagense* populations in their native habitat were frequently observed to have inflorescences with 2–3 branches (Darwin *et al.*, 2003). These phenotypic observations are inconclusive, but suggest the possibility that the *bif* truss branching phenotype is not fully expressed in *S. galapagense* due to epistatic or environmental interactions.

The literature was surveyed for quantitative trait loci (QTLs) on chromosome 12 that were detected in populations involving *S. galapagense* (previously named *Lycopersicon cheesmaniae* f. *minor*). Small-effect QTLs for fruit weight (linked to TG111 at 61.8 Mbp) and seed weight (linked to TG296 at 65.1 Mbp) were reported on chromosome 12 in a cross between *S. galapagense* LA0483 (containing the *bif* mutation) and *S. lycopersicum* UC204C, but these QTLs were a considerable distance on the physical map from *bif* (Goldman *et al.*, 1995; Paran *et al.*, 1997). An additional minor QTL for fruit pH was detected in the same population on chromosome 12 (Paterson *et al.*, 1991). However, inflorescence branching was not reported in these QTL studies, and there were apparently no other major morphological QTLs reported to be associated with the *Solyc12g019460* null mutation present in LA0483; this is agreement with the observation here that there were no major effects on plant development other than inflorescence branching and flower number when comparing the LAM183 and *bif* lines.

### *SlMAPK1* is an excellent candidate gene for *bif*

Probably due to the proximity to the heterochromatin and suppressed recombination, ~6000 F_2_ plants were required to define the map position of *bif* to a 2.01 Mbp interval containing 53 gene models (ITAG 2.4 annotation). Of those, only five genes were highlighted as having potentially moderate- or large-effect polymorphisms. Although, we cannot formally exclude *Solyc12g019130*, *Solyc12g019140*, *Solyc12g019200*, and *Solyc12g019320* as putative candidates for *bif*, these genes had non-conservative amino acid substitutions, whereas *bif* contained a null mutant of the *Solyc12g019460* gene due to the conversion of a leucine to a stop codon. This SNP was detected in three of the four available *S. galapagense* resequenced accessions: LA1044, LA0483, and LA1401 (Aflitos *et al.*, 2014), but was found in no other accessions from the other 444 resequenced genomes reported. In addition, these three accessions were each collected from a different Galápagos Island: Bartolomé, Fernandina, and Isabela, respectively (Supplementary Fig. S10); they also represent three different major clusters of 27 *S. galapagense* accessions for which a phylogenomic analysis was completed following DArTseq genotyping (Pailles *et al.*, 2017). This evidence suggests that the *SlMAPK1* null mutant is a genuine, conserved, naturally occurring variant in the *S. galapagense* clade. The truncated kinase in the *bif* line shows a 58 amino acid loss at the C-terminus of the highly conserved catalytic serine/threonine kinase domain that significantly shortens the activation loop region (Marchler-Bauer *et al.*, 2017). This major change is highly likely to abolish the kinase function and to disrupt a MAP kinase signalling pathway.

*Solyc12g019460* was initially named *MPK1*, but we adopt the nomenclature from a systematic comparative survey of the 16 tomato and 20 Arabidopsis MAP kinase genes (Kong *et al.*, 2012) where *MPK1* is renamed as *SlMAPK1.* The genes fall into four subgroups, A, B, C, and D, where the tomato and Arabidopsis genes of similar domain structure cluster separately within each group, indicating an ancient divergence of the four groups prior to speciation (Kong *et al.*, 2012). Group A includes *AtMAPK3*, *6*, and *10*, and *SlMAPK1*, *2*, and *3*. Protein BLAST results showed that the Arabidopsis protein with the greatest similarity to SlMAPK1 is AtMAPK6 at 87% identity and 91% similarity (Supplementary Fig. S6). SlMAPK1 shows 95% amino acid identity and 96% similarity with SlMAPK2 (encoded by Solyc08g014420; Supplementary Fig. S6), whereas the closest family member to AtMAPK6 in Arabidopsis is AtMAPK3 with only 76% identity and 88% similarity (data not shown). This suggests a post-speciation gene duplication event in tomato to create the closely related gene pair *SlMAPK1/SlMAPK2*; this duplication could have led to functional redundancy or diversification.

MAP kinase signaling cascades regulate many stress and defence responses in plants, but they also control many aspects of plant growth and development (Xu and Zhang, 2015); the functions of *SlMAPK1—*and of its orthologue *AtMAPK6*—have been the subject of many studies which can inform our understanding of the link between *SlMAPK1* and the *bif* phenotype.

### Is *SlMAPK1* involved in defence against pests and pathogens?

Transient virus-induced gene silencing (VIGS) has been used extensively to study the functions of *SlMAPK1*, *2*, and *3* during biotic stresses in tomato. When both *SlMAPK1* and *SlMAPK2* were co-silenced, herbivory by insects increased (Kandoth *et al.*, 2007). Also when expression of *SlMAPK1*, *2*, and *3* was simultaneously chemically repressed, there was an increase in susceptibility of fruit to *Botrytis cinerea* (Zheng *et al.*, 2015). However, neither of these two studies inform on the specific function of *SlMAPK1* alone. Gene-specific silencing of *SlMAPK2* (*LeMPK2*) and *SlMAPK3* (*LeMPK3*) did reduce the hypersensitive response (HR) to *Cladosporium fulvum*, but silencing of *SlMAPK1* (*LeMPK1*) did not, suggesting that *SlMAPK1* has little or no role in HR (Stulemeijer *et al.*, 2007). Similarly, silencing of *SlMAPK2*, but not of *SlMAPK1*, increased growth of *Xanthomonas campestris* pv. *Vesicatoria* on Hawaii 7981 tomato leaves (Melech-Bonfil and Sessa, 2011). Again consistent with the above, gene-specific silencing of *SlMAPK3* increased disease symptoms and virus content when tomato seedlings were inoculated with *Tomato yellow leaf curl virus* (TYLCV), but *SlMAPK1* silencing had no effect, and *SlMAPK2* silencing was intermediate (Li *et al.*, 2017). Thus, although the SlMAPK1 protein (reported under the synonym SlMPK6) was shown to interact with *Pseudomonas syringae* virulence proteins HopAI1 and HopF2 (Singh *et al.*, 2014), the VIGS experiments described above suggest that *SlMAPK1* has little or no functional role in resistance to disease, and this is consistent with the observation that the null allele is a common natural allele in the Galápagos Islands which perhaps would have been eliminated from natural populations under disease pressure if it was a vital component of the defence response.

### The role of *SlMAPK1* in plant development

Meng *et al*. (2012) showed that the *mpk6* mutant of Arabidopsis (null mutant of *AtMAPK6*, orthologous to *SlMAPK1*) had a more clustered inflorescence with shorter pedicels, and this was even more apparent in the case of the *er-105/mpk6* double mutant since the AtMAPK6 protein acts downstream of the ERECTA receptor-like kinases (Xu and Zhang, 2015). Studies with null mutants of *AtMAPK3* and *AtMAPK6* also found a role for these genes in anther formation (Hord *et al.*, 2008), and a dominant-negative allele of *AtMAPK6* combined with a mutant *AtMAPK3* gene gave a phenotype where floral abscission was defective (Cho *et al.*, 2008). In another transgenic study, a dominant-negative allele of *AtMAPK6* gave rise to more stomata and abnormal sepals, and a yellow fluorescent protein (YFP)–AtMAPK6 fusion protein, which was driven by the native *AtMAPK6* promoter (designed only as a gene expression reporter), gave rise to reduced apical dominance (increased branching), and shorter internodes between mature flowers (Bush and Krysan, 2007). The latter authors also noted that *AtMAPK6* null mutants had reduced male fertility, and abnormal anthers and embryos that had a tendency to burst out of their seed coats during development. Strikingly, images that bear a resemblance to the increased flower numbers seen in the tomato *bif* phenotype are reported for Arabidopsis *AtMAPK6* null mutants, including in combination with the *erecta* mutant *er-105*, or with a dominant-negative *AtMAPK6* mutant; these images showed not only more clustered inflorescences due to shorter pedicels, but also higher numbers of flowers in the clusters (Bush and Krysan, 2007; Meng *et al*., 2012). *AtMAPK6* knock-out mutants are also reported to have delayed root development and aberrant cell division, leading to the proposal that *AtMAPK6* is a regulator of the plane of cell division (Müller *et al.*, 2010), and thus potentially of many aspects of plant architecture.

The truss branching phenotype observed in the *bif* line has differences and similarities in comparison with the *AtMAPK6* knock-out mutants studied in Arabidopsis; however, the general involvement of MAPK genes in inflorescence development is clear, and evolutionary divergences in signalling pathways and different modes of inflorescence development would be expected to lead to different inflorescence-related phenotypes in the two distantly related species.

### Environmental interaction of *bif*

In agreement with previous work (Lewis, 1953; Calvert, 1957, 1959; Sawhney, 1983; Adams *et al.*, 2001), low temperature increased the number of flowers in this study (Fig. 2), but here we report a novel genotype×temperature interaction whereby low temperature had a significantly greater effect in the *bif* line than in LAM183. During the exposure of tomato plants to low temperature (15 °C), a reduction in cell division results in smaller, thicker leaves (Hoek *et al.*, 1993), and starch accumulates to higher levels (Venema *et al.*, 1999) presumably because leaf growth decreases more than photosynthesis at suboptimal temperature. This scenario might explain the greater investment of the available carbon in reproductive growth versus vegetative growth (reviewed by Van Ploeg and Heuvelink, 2005). The observation that the response of branching and number of flowers to low temperature was greater in the *bif* line suggests that a normal role for the wild-type allele of *SlMAPK1* might be to balance reproductive and vegetative growth at low temperature; indeed it is reported that the kinase activity of the orthologous gene *AtMAPK6* is activated by many abiotic stresses including cold (Ichimura *et al.* 2000), and overexpression of the closely related gene *SlMAPK3* is reported to improve tolerance to low temperature stress (Yu *et al.*, 2015).

### Conclusion

The use of genome resequencing data and an introgression browser provided a rapid method to identify the origin of the *bif*-associated haplotype and the genomic structure of the lines in which *bif* was identified. The fine mapping of the *BIF* locus identified *SlMAPK1* as an excellent candidate gene based on the presence of the only large-effect SNP and on functional studies of the Arabidopsis orthologue *AtMAPK6.* Our study provides a new locus and a positive allele for marker-assisted selection for increased truss branching and flower number for the purpose of increasing fruit yield and ripening uniformity in small-fruited cultivars (e.g. cocktail or miniplum types). The literature suggests that the *SlMAPK1* null mutation should have little or no effect on plant susceptibility to disease, although further work to test this possibility, and to look systematically for wider effects on plant development, is required. The proposed interaction of *SlMAPK1* with low temperature and its potential mode of action through regulating the plane of cell division suggest further studies to understand the role of MAP kinases in mediating plant architectural plasticity.

## Supplementary data

Supplementary data are available at *JXB* online.

Protocol S1. 96-well DNA extraction with Chelex-100.

Table S1. SolCap markers used for genotyping.

Table S2. Additional SNPs used as KASP markers.

Table S3. Linkage of KASP markers to *bif.*

Fig. S1. Pedigree of seeds used and their accession numbers.

Fig. S2. Schematic diagram of the regime used in the cold transfer experiment.

Fig. S3. Genome-wide SNPs in *bif* and LAM183.

Fig. S4. Genome-wide InDels in *bif* and LAM183.

Fig. S5. Tissue-specific expression patterns of genes in the *BIF* mapping interval.

Fig. S6. Genomic DNA, cDNA, and protein sequences of *SlMAPK1* alleles.

Fig. S7. SNP similarity map for chromosome 12 (87 accessions).

Fig. S8. SNP similarity map for chromosome 12 (31 selected accessions).

Fig. S9. SNP similarity map for the *BIF* mapping interval (87 accessions).

Fig. S10. Collection sites for *Solanum galapagense* accessions.

Supplementary Protocol Tables Figures

Supplementary Figure S7

Supplementary Figure S8

Supplementary Figure S9

## Data repository

Data underlying this study can be accessed through the Cranfield Online Research Data Repository (Silva Ferreira *et al.*, 2017). DNA sequence read data are available via NCBI BioProject accession PRJNA378916.

## Abbreviations:

BIF: bifurcate flower truss
FM: floral meristem
IM: inflorescence meristem
InDel: insertion or deletion
KASP: Kompetitive Allele Specific PCR
SAM: shoot apical meristem
SNP: single nucleotide polymorphism.
