# Supplementary material for: BIFURCATE FLOWER TRUSS: a novel locus controlling inflorescence branching in tomato contains a defective MAP kinase gene
Source: J Exp Bot. 2018 Mar 2;69(10):2581–93. doi: 10.1093/jxb/ery076 (PMC5920302; doi:10.1093/jxb/ery076)
Supplement: Supplementary Protocol Tables Figures [file ery076_suppl_supplementary_protocol_tables_figures.pdf]

SUPPLEMENTARY DATA

Supplementary Protocols

Protocol S1. 96-well DNA extraction with Chelex-100.

A) Preparation

1. Prepare 5% w/v Chelex-100: add 2.5 g of Chelex 100 (Bio-Rad, Hemel Hempstead, UK) to a 50 ml Falcon tube and make up to 50 ml with freshly obtained, deionized (MilliQ) water (autoclaving not required).
2. Add two 4 mm soda glass balls (Smith Scientific Ltd, Kent, UK) to each well of a 96-well deep-well plate (Starlabs, Milton Keynes, UK).
3. At room temperature, add a small piece (~20-30 mg) of young leaf tissue (e.g. small developing leaves from the apex which are less than 15 mm in length) to each well, placing it on top of the glass balls.

B) Tissue disruption and DNA extraction

1. Add 300 µl of 5% w/v Chelex-100 to each well.
2. Seal the plate with a sealing mat using a seal applicator, or with a disposable adhesive seal (Starlabs).
3. Disrupt the tissue for 30 seconds at a frequency of 30 Hz using a Star Beater mixer mill (VWR, Lutterworth, UK).
4. Transfer 100 µl of supernatant to a PCR plate (Starlabs) and seal the wells using 8-strip PCR Caps (Starlabs).
5. Incubate the plate at 100°C for 5 min in a PCR machine (MJ Research, Canada) with a heated lid to prevent evaporation.
6. Centrifuge (5430R, Eppendorf, Stevenage, UK) using a swing-out rotor at 3800 rpm for 4 minutes at ~20°C.
7. Plates can be stored at 4°C for short-term (< 1 month) or frozen at -20°C for longer term. If using after storage, re-spin to bring down condensation and ensure the pellet is secure at the bottom of the tube.

Supplementary Tables

Table S1. SolCap markers used for genotyping.

Marker ID	SolCap Reference	Chromosome	Position	SNP
DSF1	solcap_snp_sl_59771	1	6229659	T/C
DSF2	solcap_snp_sl_16925	1	18519338	T/G
DSF3	solcap_snp_sl_19068	1	43255975	T/C
DSF4	solcap_snp_sl_22423	1	59436302	C/G
DSF5	solcap_snp_sl_17075	1	82722469	A/G
DSF6	solcap_snp_sl_17448	2	15153844	A/T
DSF7	solcap_snp_sl_6255	2	20334143	A/G
DSF8	solcap_snp_sl_13842	2	29361519	T/C
DSF9	solcap_snp_sl_67542	2	35723186	A/G
DSF10	solcap_snp_sl_100592	3	7100090	T/C
DSF11	solcap_snp_sl_1779	3	50094991	T/G
DSF12	solcap_snp_sl_21694	3	60059066	A/G
DSF13	solcap_snp_sl_20714	3	67127672	T/C
DSF14	solcap_snp_sl_2071114	4	5020084	G/T
DSF15	solcap_snp_sl_101013	4	54543361	A/T
DSF16	solcap_snp_sl_69262	4	60067355	C/G
DSF17	solcap_snp_sl_47590	4	64717474	T/C
DSF18	solcap_snp_sl_51106	5	8917895	T/G
DSF19	solcap_snp_sl_51600	5	20576788	T/C
DSF20	solcap_snp_sl_69404	5	52116995	T/C
DSF21	solcap_snp_sl_12268	5	63293808	C/G
DSF22	solcap_snp_sl_34975	6	3502385	A/G
DSF23	solcap_snp_sl_36705	6	32122683	T/G
DSF24	solcap_snp_sl_55874	6	37730097	A/G
DSF25	solcap_snp_sl_54417	6	48367262	A/G
DSF26	solcap_snp_sl_11180	7	1815826	A/G

Marker ID	SolCap Reference	Chromosome	Position	SNP
DSF27	solcap_snp_sl_38939	7	57109419	A/G
DSF28	solcap_snp_sl_55505	7	63665011	A/C
DSF29	solcap_snp_sl_7305	8	711380	A/C
DSF30	solcap_snp_sl_56732	8	1916037	T/C
DSF31	solcap_snp_sl_4374	8	55937442	T/G
DSF32	solcap_snp_sl_34862	8	63766185	A/G
DSF33	solcap_snp_sl_28404	9	651775	A/T
DSF34	solcap_snp_sl_45095	9	6096766	A/G
DSF35	solcap_snp_sl_29222	9	67465994	T/C
DSF36	solcap_snp_sl_69743	9	70776666	T/C
DSF37	solcap_snp_sl_45992	10	49125	T/C
DSF38	solcap_snp_sl_46021	10	162566	T/C
DSF39	solcap_snp_sl_16511	10	58189616	T/C
DSF40	solcap_snp_sl_8834	10	64340314	T/C
DSF41	solcap_snp_sl_62736	11	5151004	T/G
DSF42	solcap_snp_sl_732	11	10015478	T/C
DSF43	solcap_snp_sl_2996	11	30715391	A/G
DSF44	solcap_snp_sl_53061	11	50649946	A/G
DSF45	solcap_snp_sl_41168	12	3036369	T/C
DSF46	solcap_snp_sl_16795	12	10579861	T/C
DSF47	solcap_snp_sl_59087	12	44105019	T/G
DSF48	solcap_snp_sl_53957	12	62088020	T/C

Table S2. Additional SNPs used as KASP markers on chromosome 12. SNPs were chosen from the SolCap markers where indicated, or were specific to this project where no SolCap reference is indicated. The SNP causing the gain of stop codon in *SlMAPK1* is shown in bold (marker DSF72).

Marker ID	SolCap Reference	Position	SNP
DSF50	solcap_snp_sl_41220	3240286	T/C
DSF51	solcap_snp_sl_32654	4777800	T/C
DSF52	solcap_snp_sl_63506	5833035	T/C
DSF53	solcap_snp_sl_20409	7479839	C/G
DSF54	solcap_snp_sl_40598	8948057	A/G
DSF55	solcap_snp_sl_52407	45654100	T/C
DSF56	-	51569050	G/A
DSF57	-	55725286	G/A
DSF58	solcap_snp_sl_42961	59225471	A/T
DSF59	solcap_snp_sl_53990	61861142	T/C
DSF60	solcap_snp_sl_53957	62088020	T/C
DSF61	solcap_snp_sl_16796	11159684	A/C
DSF62	solcap_snp_sl_53090	25928732	T/C
DSF63	solcap_snp_sl_38520	38590425	A/G
DSF64	solcap_snp_sl_52402	45339818	A/C
DSF65	solcap_snp_sl_18995	47510753	A/T
DSF66	solcap_snp_sl_52417	48203620	A/T
DSF67	-	50481042	C/G
DSF68	-	8566567	A/T
DSF69	solcap_snp_sl_40598	8948057	A/G
DSF70	solcap_snp_sl_16794	9973851	A/G
DSF71	solcap_snp_sl_16795	10579861	T/C
**DSF72**	***SlMAPK* L291***	**10389589**	**T/A**

Table S3. Detection of KASP markers linked to *bif*. Twenty three individuals from a population of 96 LAM183 × *bif* F_2 plants with the *bif* phenotype were selected. A Chi-squared test was used to test the null hypothesis that KASP marker scores segregated with a 3:1 ratio in the *bif* plant population. Low *P* values ($P < 0.001$) indicate linkage between the KASP marker and *bif;* these are highlighted with bold text and grey shading. Chromosomal positions of all tested markers are given in Table S1. Note that some markers given in Table S1 were rejected due to technical failure or were observed to be non-polymorphic and are not shown here.

Chromosome	Marker ID	*P* value	Chromosome	Marker ID	*P* value
1	DSF1	0.383	7	DSF26	0.908
1	DSF2	0.238	7	DSF27	0.806
1	DSF3	0.190	7	DSF28	0.637
1	DSF4	0.190	8	DSF29	0.785
1	DSF5	0.912	8	DSF30	0.121
2	DSF6	0.529	8	DSF31	0.204
2	DSF7	0.529	8	DSF32	0.108
2	DSF8	0.529	9	DSF33	0.683
2	DSF9	0.606	9	DSF34	0.113
3	DSF11	0.606	9	DSF35	0.691
3	DSF12	0.460	10	DSF37	0.346
3	DSF13	0.059	10	DSF38	0.399
4	DSF16	0.821	10	DSF39	0.273
4	DSF17	0.785	10	DSF40	0.739
5	DSF18	0.279	11	DSF41	0.663
5	DSF19	0.279	11	DSF42	0.302
5	DSF20	0.547	11	DSF43	0.302
5	DSF21	0.174	11	DSF44	0.346
6	DSF22	0.691	12	DSF45	0.166
6	DSF23	0.691	**12**	**DSF46**	**0.000162**
6	DSF24	0.123	**12**	**DSF47**	**0.000481**
6	DSF25	0.806	12	DSF48	0.233

Supplementary Figures

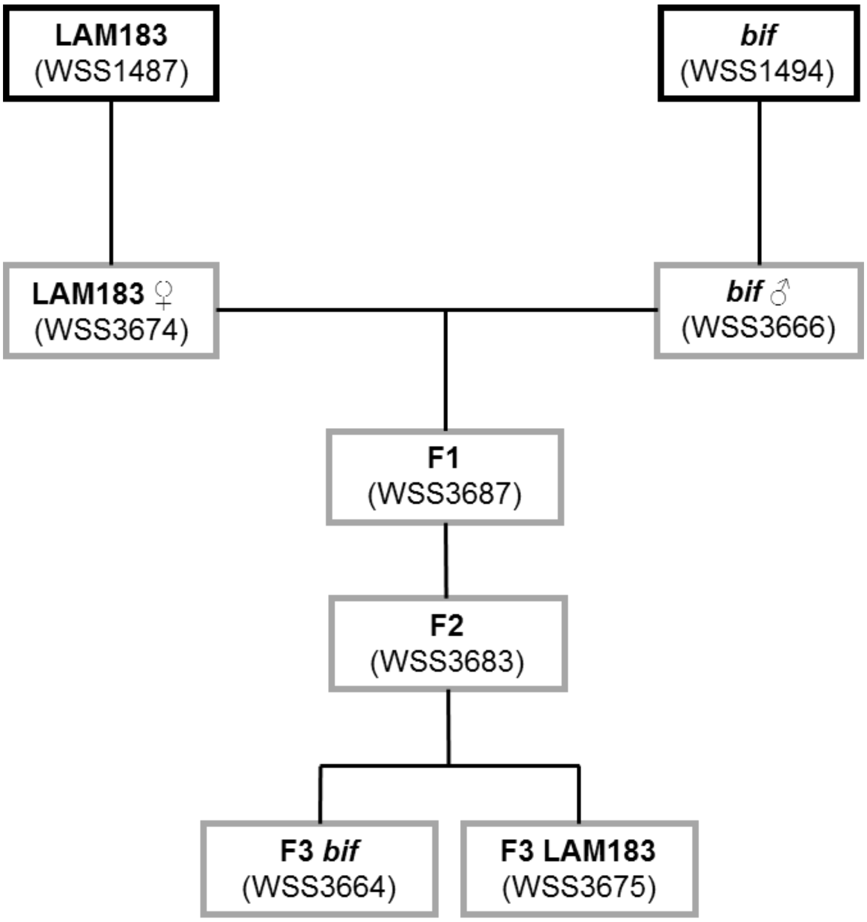

Fig. S1. Pedigree of seeds used in the experimental assays. F_2 seeds used for fine mapping were from seed package WSS3683.

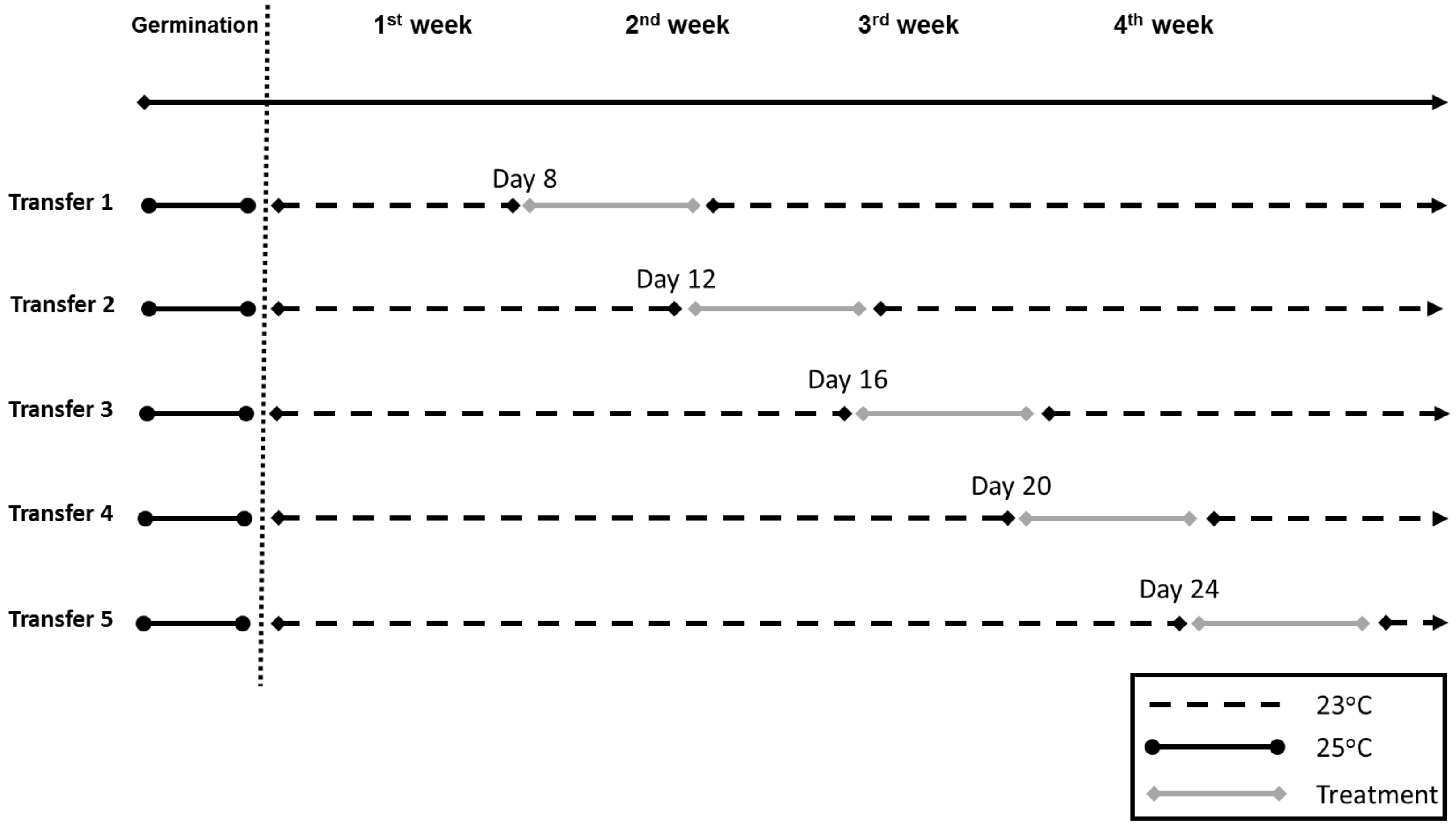

Fig. S2. Schematic diagram of the regime used in the cold transfer experiment. Plants were transferred from a glasshouse with a minimum set-point temperature of 23°C to growth cabinets set to either 15°C or 23°C (“treatment”) for periods of four days and then returned to the glasshouse.

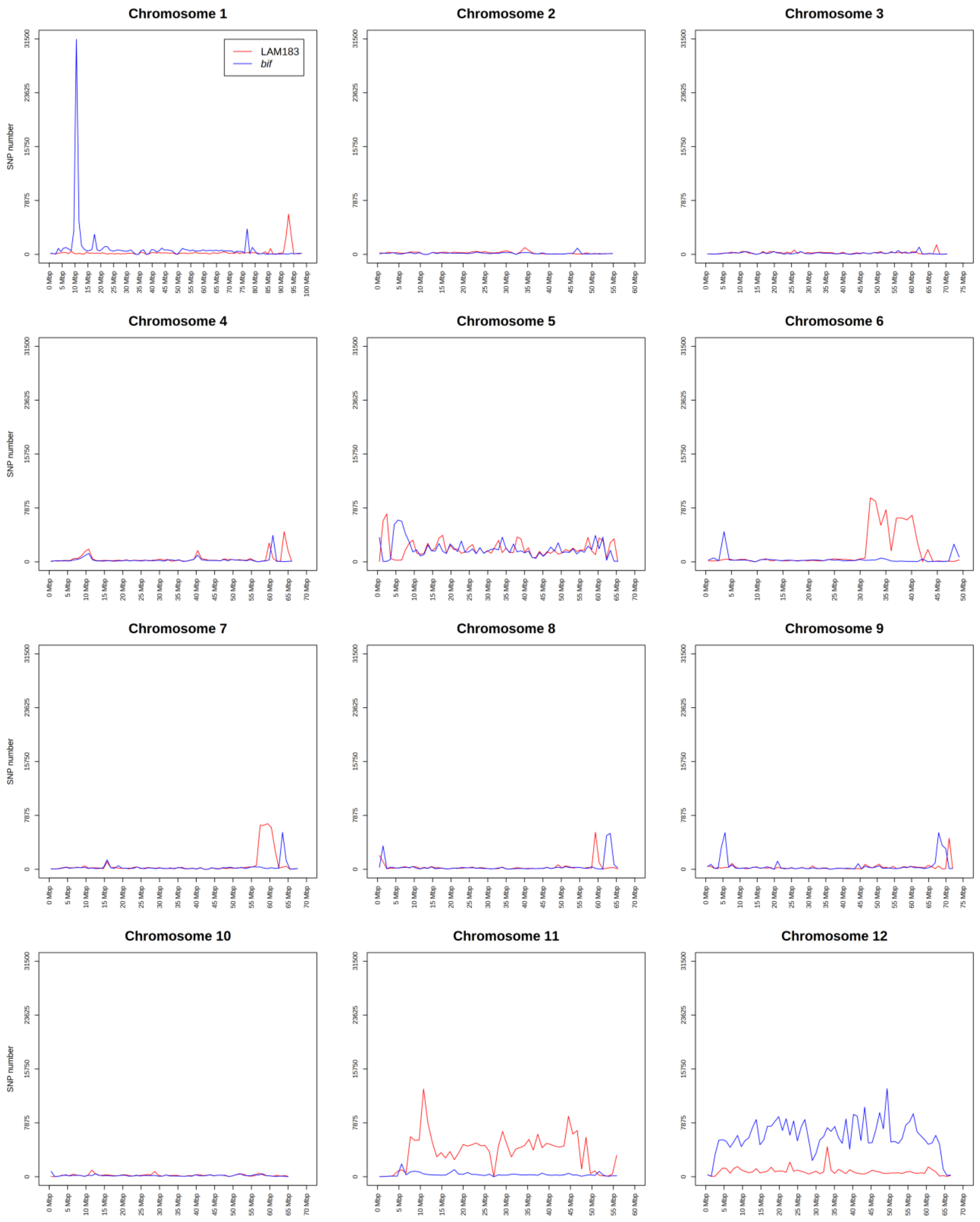

Fig. S3. Genome-wide SNPs in *bif* and LAM183. The number of SNPs in comparison to the Heinz 1706 SL2.50 reference genome was plotted for each of the two lines using bins of 1.0 Mbp. SNPs that were non-polymorphic between *bif* and LAM183, or that had a quality score of < 230.8 were excluded. The number of SNPs that are polymorphic between *bif* and LAM183 can be obtained by summing the two plots. The *x*-axis represents chromosomal position in Mbp.

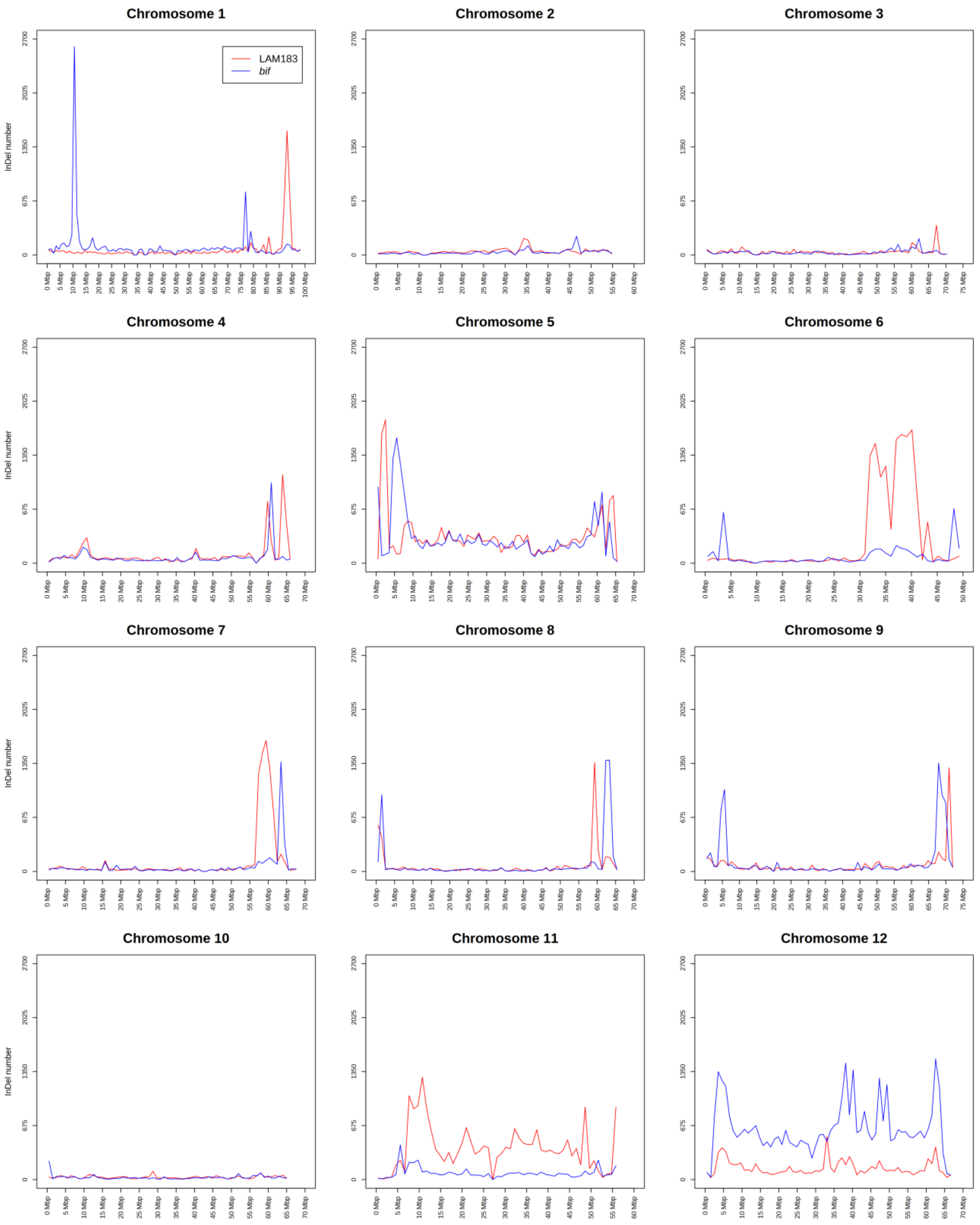

Fig. S4. Genome-wide InDels in *bif* and LAM183. The number of InDels in comparison to the Heinz 1706 SL2.50 reference genome was plotted for each of the two lines using bins of 1.0 Mbp. InDels that were non-polymorphic between *bif* and LAM183, or that had a quality score < 230.8 were excluded. The number of InDels that are polymorphic between *bif* and LAM183 can be obtained by summing the two plots. The x-axis represents chromosomal position in Mbp.

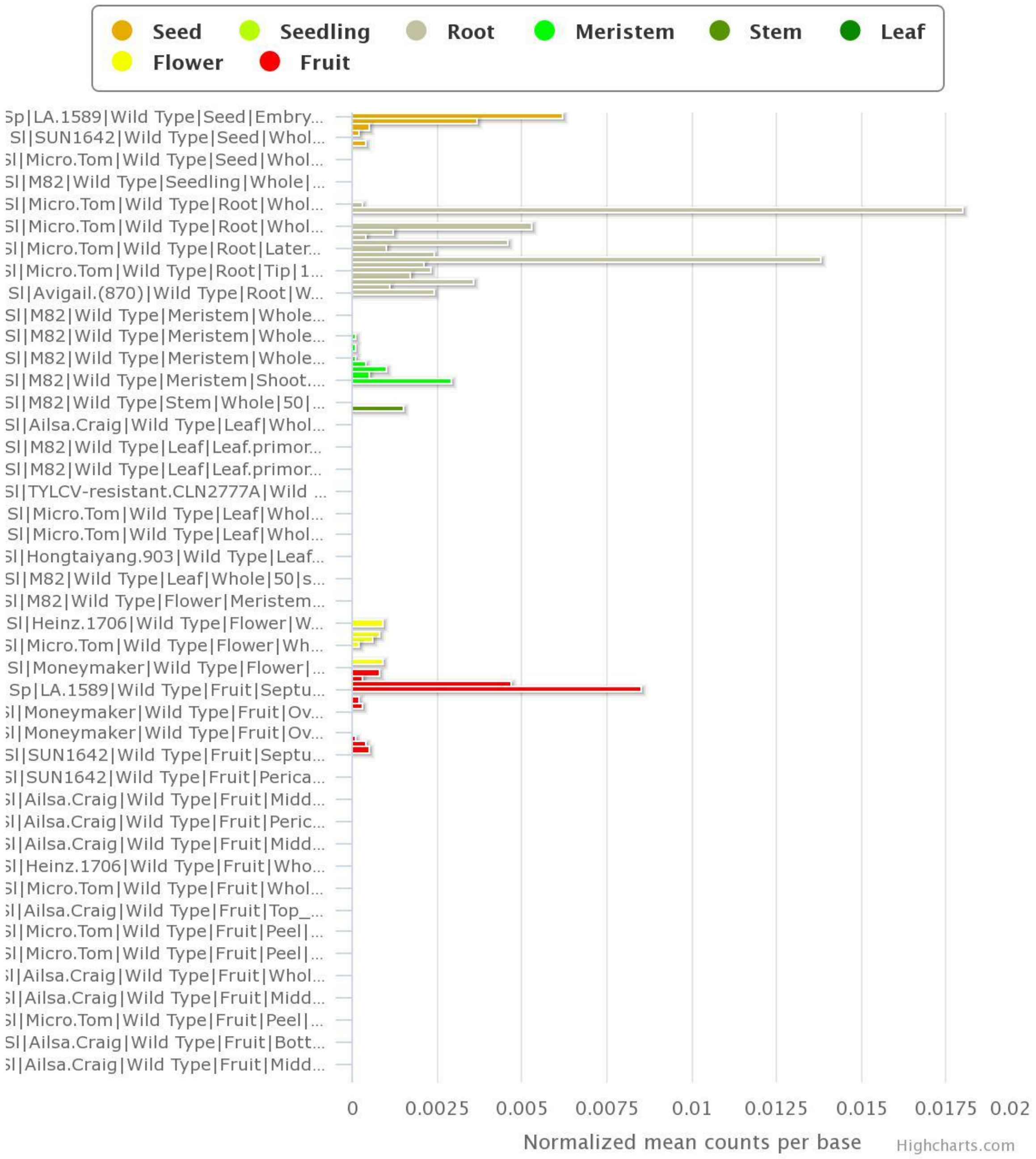

A: Solyc12g019130

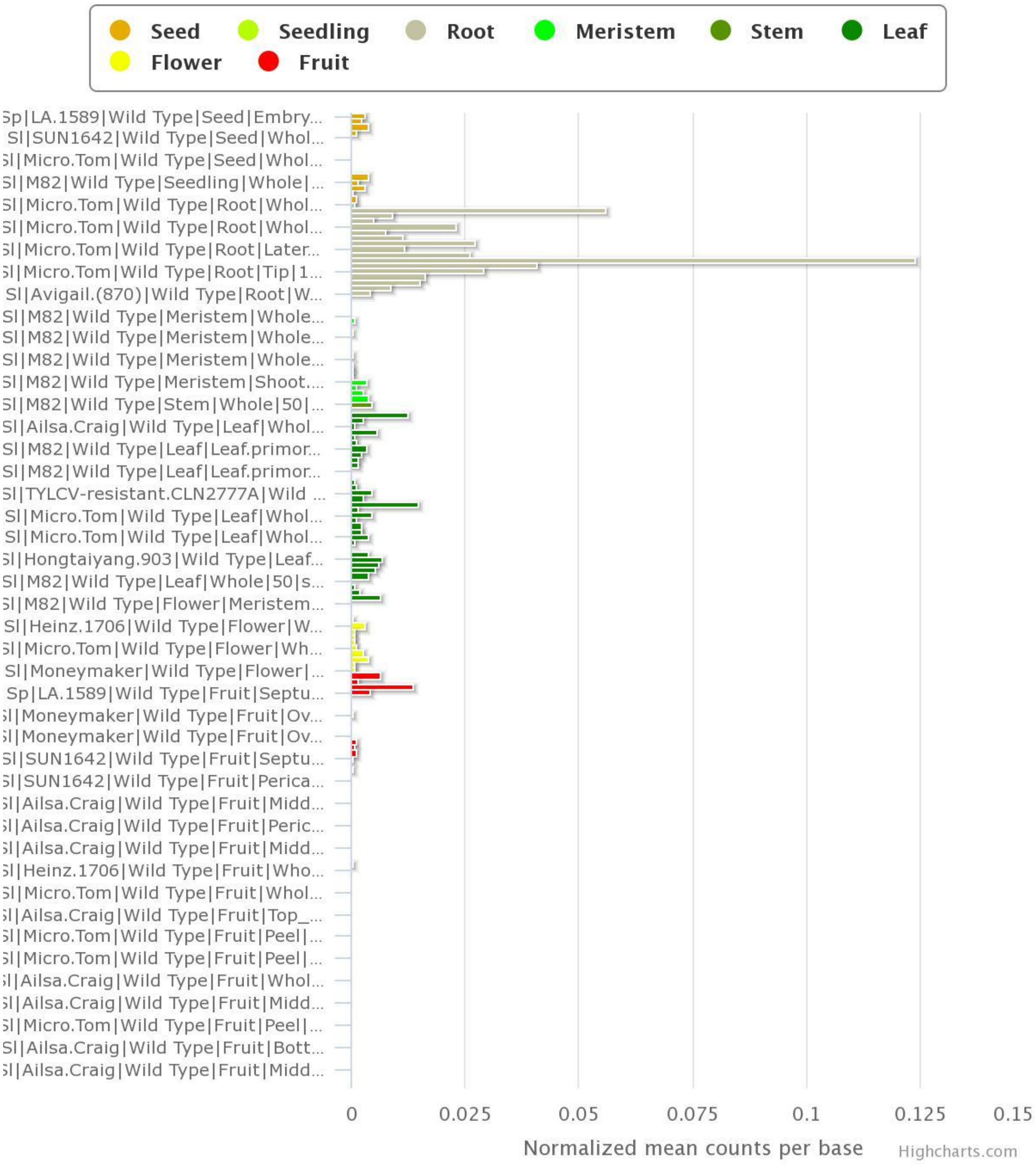

B: Solyc12g019140

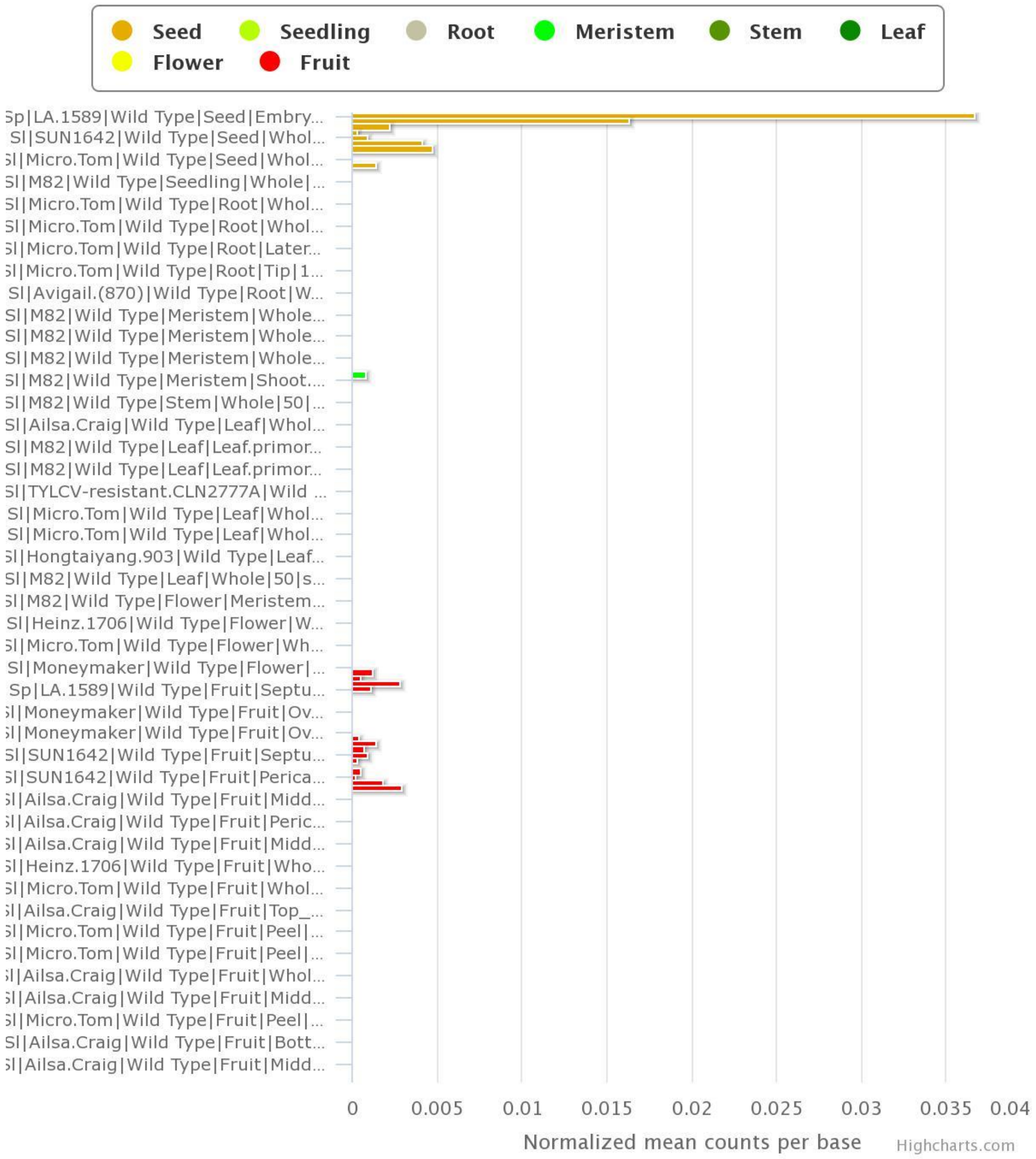

C: Solyc12g019200

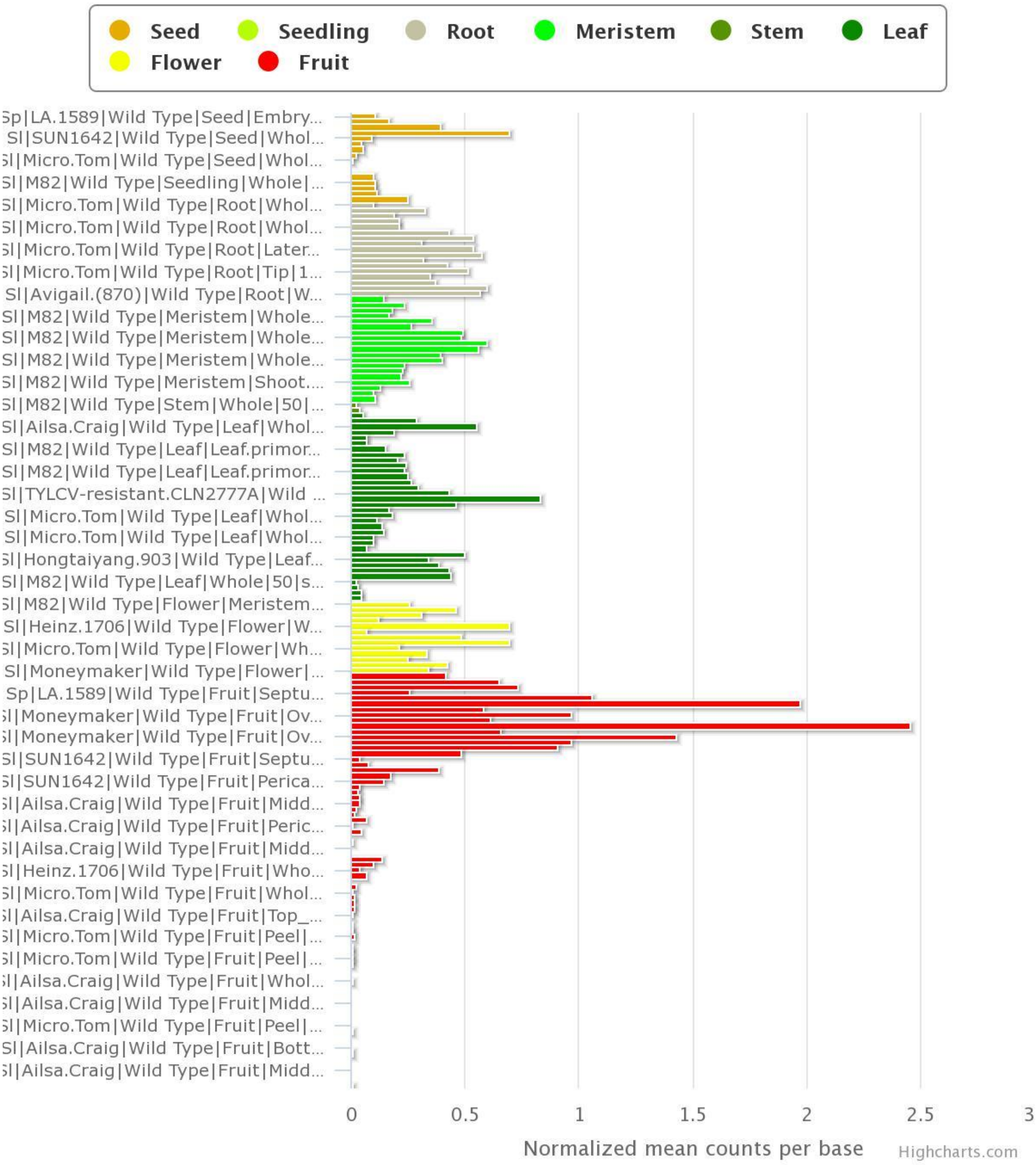

D: Solyc12g019320

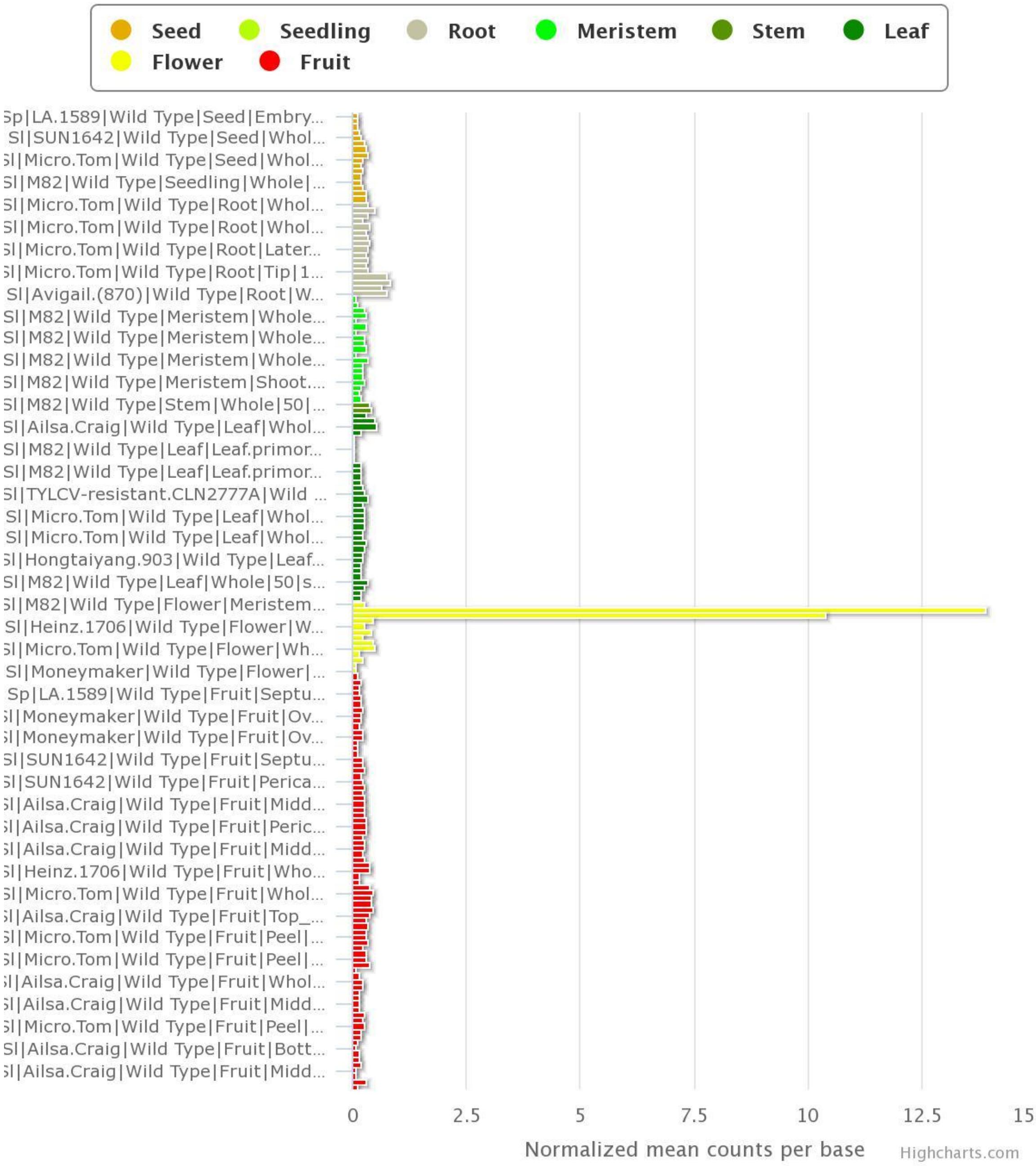

E: Solyc12g019460

Fig. S5. Tissue-specific expression patterns of genes in the *BIF* mapping interval. Five genes (A to E) were selected based on the presence of moderate or large effect SNPs between LAM183 and *bif*. Normalised mean counts per base are shown for each tissue sample. Data and downloads of plot images are from the TomExpress database and website (http://tomexpress.toulouse.inra.fr/) representing a collection of public RNAseq experiments. Colours indicate tissue classes. Locus IDs are given under each plot.

A: Genomic sequence of *Solyc12g019460* (*SlMAPK1*) from Heinz 1706. Note that exon sequences are identical between Heinz 1706 and LAM183.

ATGGATGGTTCCGTTCCGCAAACGGATACGATGATGTCGGATGTGGCTGCACCTCCGGCTCAACAACCACCTCCG
CCGTCACAACCGCTGGCTGGAATGGATAATATTCCGGCGACGTTAAGCCATGGTGGCAGGTTCATTCAATACAAT
ATTTTTGGTAATATTTTTGAAGTTACTGCTAAGTATAAACCTCCTATAATGCCAATTGGTAAAGGTGCTTATGGA
ATCGTTTGgtgagtttctttttctttcttttgattactttctatcaattttaaaaactacgctatttctcaat
ttactacttgtaacgtatttttgggtttttttttgtgtgttgattaatgattttgactttttttttttatttga
ttgattgtgtgtag**TTCTGCTTTGAATTCGGAGACAAATGAATCTGTAGCAATTAAGAAAATTGCTAATGCTTTT**
GATAACAAGATTGATGCTAAGAGGACTTTGAGAGAGATCAAGCTTCTTCGACATATGGATCATGAAAATgttagt
gagttacctgagtatgtttaactgtgttagtttattttcgtatgaatctggatagtagaaagagtaggttttggg
tgtaatcacatgctcgaattcagttagttcaaattaacttaattaataagaacagaatattacatttgacttttt
gctgagagaagggcttagatgattttgttttcgtttctgcaaatggtaagacagatagtgtttttttttttgttt
aatgtcatggtacagaacatgtttaatggcgttcatgcatataagtatggaattgttttcgtttattatatagag
gacaaattgtatttttgtctctcggattgtaccgtctgttggatatagatgttttcaagatatttgtggcttcttc
ttgaggatatttgaggaggctattgagaaaaggaaaatgagtctgtataggcttttgttgttggctaatgtgaca
tctgtcaatgcag**ATTGTTGCGATCAGAGATATAATTCCACCACCACAGAGAGAAGCCTTTAACGATGTTTACAT**
TGCGTATGAGCTTATGGATACTGATCTCCATCAAATTATTCGCTCGAATCAGGGTTTATCTGAGGAGCACTGCCA
Ggttagaatcagaacatttaattcttcaaacaacaatttaaaaacacaactaaagtgtgagtacccgatgtgttt
ctcaaagcttgtttctttttttttccgcttcctttttctggttaaaaaatgaatataggaaaaaaaatctcatgt
ttcacgacatattcatcattttagttgtttaaaccacataacttgtttggaagaattcttctgactggcctgca
actaatttcatgtgtgaactagtgcaggttgacaatttaaatgatttaatttttgtcagaacaacgtgtggccag
aaaattggtttctaagttttaagaataggaaatgaaaatttcttactgagaagtggaaatgttctattttgtaca
cgttgtacttttgtatgctaagaaatatgtgatagcatttaccttctttcctcttatggaatcttattcggcttc
ttttctcaccaaataatgctcatggaaggacaaaacttaaagtagcttacagttggcttgagattacttttagat
tgagaattctgactagcctgagatgttatgcacttccgaagagaaatataattttttttcgtaaaatttactttg
tgggtagcagggtaggtctgcctcagtggaataaagttattgtcaacttactgaaaagtaacaaataattaggta
ctgttccatggattgttcatttatgttgacaagctcatggtgaacgattataatgttggtatattaactttataa
gatcttccaaagttaccttagagattaagaattgcatatacaattgtaagttacatactcatattatgttcttat
agaacatacaagtgtaagttacatactcatattatgttctttacatgcaacaaagaactctcaacctctaaaata
accgaacttcgttagatttattgaactttaagatgtaacattaaattcctaggagaagagccagaggaattcagg
aaattacccacaatggtccatgataaaatttcaagagaagtagattttaccataagaatgttaacgctaacaaca
acagcataccagcataatccacaagtgggggactaaggagggagggtgtacgtagaccttacccccgaccttgt
ggggtagagaagcaattccaatagacccttagcttaagaaaatcattttatagaacaaatttgaaaggagtgcca
aagtagaatgttatgatgaacagtgaagaaagataagtattgacaatagatatagtagaataacctaagtaaaga
aacaacaatagtaataaatttgatgaataagataatgctagcataataataatattagcaatattggttatggaa
tagaaaggtgctccataataccattcagaaggggagaacacttgactacctactaatcatttgccctaatcctct
accttcatgctctcctatttatcgtcatgcgtgttgtgctctgtcaaaggacctctctcctattcttcctcaggt
tacctctactgaaaacctttagactctagggtctgtctccatgcatcttgtctatcttaactctgctgcatgtct
catcgatcaatacaactactgaaagagttttaatttgaatagattaaagaatggaacttgttgcgattgtgagac
tgcatgttgcattgcatcaaatccaatgttttagaagaggaccaaacacatagagactaactgtcattttgtcag
aaagaagaaagaaggtactctacgaagacatgttaatataactatttaaacccaatagcttgagggaataattga
gttgagttctctcgtattttctcttttacttcacaacttcaatatagaagaacaaaaaacatgaacaaacaccat
gaatatgtctatacaacttgtataaaaagtttcttcatcaaaagaaaaaaaacttgtataaaaggttatttggtt
caggttttaaaaaaaatgcaaaaccaaaaaaatattgtcagtttattataattggaaaccaaaacaaaccaacta
taataatgtctgtttggtttgattttcgatttgatttgatttttttgtttttcatgaacacccgtaatagtaatag
tgcatattgtgctacttagattataatgcatttccgcaccccatttgtggattacactgggtgtattgccgttga
gtttataatgcattatcgtgtctaaatttgtctgactaaaactaaatacttctccatttgggtgcag**TATTTCTT**
GTATCAGATCCTCCGTGGGTTGAAATACATACATTCTGCAAATGTTTTGCACAGAGACTTAAAGCCTAGCAATCT
TCTCTTGAATGCCAACTGTGATTTGAAGATATGTGATTTTGGGCTAGCTCGTGTCACTTCTGAAACTGACTTTAT
GACCGAATATGTTGTGACAAGATGGTATCGTCCACCTGAGCTGTTGTTGAATTCATCCGACTATACTGCAGCAAT
TGATGTATGGTCAGTGGGTTGCATCTTCATGGAGTTGATGGACAGAAAACCCCTCTTCCCTGGCAGAGATCATGT
ACACCAGCTGCGTCTTATTATGGAGgtatctattgttcactatctaccccctgccccccttacactaaagaagtga

gtttcctaagtttttgttcttttatctcatttttcgtatttcacctctttgtactggctgttttgtgcaaatcca
tttaaaagaaaagagtaacatcaagttgtagatctgggaaacttaatggagaagccttagacagagtgtagtttt
atttttgaaccgttgcttctctacgtcaagaattacaactgatcaactatgctttcttctcagcaagtgttatgc
acatatgtagtatgtgatcttaagaaggaaaacaaatttacctgccactctctttcagtctttcctataagaaga
tgaacaatgcatgattattttgtattatgaatatatagtttttcatagtaatggcccgaatctcttttattatct
tacattatgctcaaattaatagatgtgattaatacttaaatttattatgatgaccgcaacttcatgcag**TTGATT**
GGCACTCCTTCAGAGGCTGAAATGGAATTTTTTAAATGAGAATGCAAAACGCTATATCCGACAACTTCCTCTTTAC
CGTCGACAATCATTTACTGAAAAGTTCCCGCATGTAAACCCAGCTGCTATTGATCTTGTCGAGAAAATGTTGACA
TTTGATCCCAGAAGGAGAATAACAGgtgcgaatttccagactagtttttctcatagctgcattaatcaattctct
atctgcatcttacactcttttcttgaactgtacaaatgttcacttgttttattgcaagagatcaatcaattattc
tctcctttttttctcatcaatgaaggaaaacagaatctatttcaagtaagtattttattgatgtgtagcaaaaaaa
aacagcatttaggaaccgtggatgagaagtacatctgaaagagaattttactatgtatgtaaaagagccaaaaga
attcaacaatcatgcctggctctaaaacataattcggaccttcgttgaaccaaaatctaagcaatgctatactct
ttagcttaaatttaataatgtttcccttcttgtcttcaaaaactctagcattcctctctcgcaatacgacccacc
aaacgacagcagaattgtgagtccctgatgtttttcggcatacaccaattcaagccccaaatgatttgaaaagag
atgctaaatctgttgggtaaacttgcttatcaatggtgagacatcttccttgggctacaatctaagatacacact
tgatcttatatagaaccagattcttcaatgaaatcttccaaggccaggagacatagttgggaaggggttagtcaa
gtctgcataatagtagagagaattccattttgggagtgtttccagatgacggaatctagctgagagatcagagga
gggacagggttttgctgattcatcatagcacaaaattcatcaattgcccaatcattgaggtttctcctacatgga
atgagccatccttgatgtggctgaagttcggttgatacagagtcttggttggattcatatctaaaacgaaaaggg
aatctgctcatgagaggggtgtggccaagccacgaatgttgccaaaatctaatctttctaccatcacctactcta
aagaacattgttcccaagtctaggttcgaagagcaacatcaaagagttatcactaagcattacgaagttgctatc
attagagttgacgattaagcaatataccccccatggcttgggaagggccataaataagtaccaacacaaaccaatc
ttagcttgaaaagtatgggaacatttaaccccgttgtttgccaagttggtgactttagatctcttgaaccttcac
aaagaatccaagaaagtaagctgggaccccttcattgcaatcgaccttatccttaagtacttcctacatttgtgt
tacctgttcaatgtaaacccatcttgttgattgttaaatcaaaggcaaacccttttctctatatgctttcttta
ttccttcttctctttgaaacttaggaaattctctcttcattcccaacttgctttcccttaacaatgtcacatcta
gtgaactcaactagacttccctgagcaacaaaatcaactggttgctccatataaacttcattctcaaggtcagcg
cagaaaaaatcattcttgatttccaactgataatgaggccggcggaggacaacaactatgtattgataaagatgg
aaaaatgcttagccacaatagagaaagtatcactataatcaagcctaaatatctgagtatacccattgggaccaa
ggctggccttaaccaattaacatggtcactcgtaccaattttgactgcataaacccaactacaaccaacaataga
tttatgaagaaaggagaacaagctcccaagtatcacttgtgtgtagaacaaatgttctgtcatccttgatgagaa
aatgcatcactcgtagatttaggaatggaaatagaagacaaagatgatacaaaggcatagtggggtgatgactga
ctgtgataacttcaaaatatataatggggattagtattaccagcggattgtatacccttttggagtaaattggtt
gattaagaggagataagtctgcagtaggcacagggtttggcataagaggtgaatcatctgagcttgatgctggac
gtgaatgacgggaagttatgattggtggctggagatgtagtatgttaaactttgtaaatattaggatgataaaat
aagatgtatattaaggtcgagactaaatatgtgacctcgaccgacttaaggtaccaacaaagattaggtgagtag
taatgatacctcttttgatctccactgtttatcatttcctccttccaaactctttttttcccataacactaatatc
cccatcacttcttccattagcctctaaaatggacttctctcaaccatctgttgttccagatttgtcggtagagat
ttggattagtacttgttagcttagtttcaaccaacacatgaatgtcagccggcccactgaagcaacagatttctt
acaagtgttcttttgctctccccatttaaccctcttaacattccaagataaaaaaaaattatcttcatgataaac
ccttgtgtgagattctccctcttgttcttggctccccttctttgaatttcacatcaaaagccaaattcctaaatt
ttaggaattactgctgtgagattatcctgcttgtcattcctccttttgttttggtccatccccatcctttgatcg
atccttaatagcaattcatatgctatgtcctcacaacccgcaaaagctgcaccaaatttttttccctaatttgatg
atatttgagtgtatccacaatgttgctttctccttcacttcttcctcaccgatcgattcgtgcacttgtagtggc
tctatctcattaaaatttatcatctactttgggttactctcctgggttccttctttttcccaaggttgaatttcc
atggagctacttccaactatgttcttttcaccgtgaagaatatggatttccatgaaatgcatctttcctctctct
gcttttctgttatgattctgatagactcctgttcatcagcaaagatgcggaaatcctgattttgtcccgtactct
ttggatgagtatggatattatctatgaaaggctcagccgttttaattactgagggctttggtgacttgggtttaa
aaaaaacttaatactttgtccttcagctgcccctggcctgcctaagtctttggccagaataatttagtccactag
tgctaggtgtctccattggcccaacctatgtggaccctctcccacatgtcaccccccctgcttctatcctacgtgc
cttcaatctccagctcctcaatatccattggatatattccagccctccttttcccttcagagtgccgagctgact
atctaccttattctggctcttgtcactctcatctcgatggtcaagcttgcgatcattgaccttttgtatcttgcc

agagcttctacccagatcggtggaagaacactatgtcaccatctgcgacctctacaaagcatggaatttccttga
gaggtcccttcgccttggtgtgagcccatctcatatggttcttgagtagcatttcttcttctgcctctaaccaac
caccgcacttgtcccctatttctttatcacgtgttctgatcagagctcaagtgggatcccaagggctcttatcc
agaaagagtcacattttattggatggataagcccctgttgttggagaccaccactggactttcaagctcatccg
ctgtctcatccatctccccccagaatgtgttcagcgatctttcgagactgggtggttatggacccctttccacga
ctgttctacccattccttacatcgttaagggtggggatctcatcatactctggaaattacgcactaggaatctgc
ttaggagttcatttctgcctttggatttgcaggcatcaagagagttaacttcctgctgtgtctcttgttactttt
gggcccatatgtttctgtgtattgcttctttatagttctttttctcttttgtgtattagaatgtatactctcgtt
ggcatttaacacgagctgtgtatttggaccatttatgatgccttcaattttgttgcaagccccaacaaactcctt
attacagctgttctccggcagaattattactgatctggatgaaccatttactccaatcatatatatgaatctacc
atatttattgaacctaaaggagaagcgaaaataggtaaccagatctctgcctctccatgatttaaaagctttccc
ccttgtgtctgatgcctctcgcagcctcttgactaaacaaggctacctaactaagtgtcatccttcgcgcgtgga
gctttgattctccacccagtcgaaccaagttccgttagaggattttgtctggtttatatcatatgatttacacc
ccacccccacccccaagtctgacataaatacggttctccattagaggatgggttctggtggtcaaataccaact
gaagtattgtctggaaagtcctagaggggctctaagaagccctgcctaggaactcatgctagggagcatttttat
aatatattatttttttgcatcctgtgacctaagtgatcctaaattatgtacaaatatcagtcaatattctgagatg
ataagaaccctagctcctttttttacaaaatcagtaactaacctggaagctaagagatgaaaattgagcttaatg
ggaaaaaacaaggcggggagaagggtttagaggcacaaaccacagcggaaccatggcccgaaaggaagagaccca
attttgattttaacagatgacccccttaagttgttaacctatttctgtttctttttatttctgctagatggaaat
atatttccatgtgaagtgccacatgaacacccttttttaacataaaaaggagtgtaccacacaccatcaacattt
catctaggtttgttttaatctatgttgctcaggctcttcaaaaatgtaatttttggagaatcgacatgggtgcgg
catcagaagtgaagaatctgtgcaagttaggttttaatacatatataggatagtttaggtagggtataaggaacg
agtagttggggattgaaatttgcgaaaaactgatagttcatgtgtttttgacgaaaaaacagctcgttgagcttc
cgagaatggggggattttcagcagttgctcaaaccggatctttcagtggccaactgtaagatttcaactcaaaatt
gaggcgaacaatctatttactcgatagaactcacaatagaattccaggaagtttcagagaggtttaaaccaataa
caaaataagaatagtgaagaatgaaggacgtgcatggtttttctattttgggatttttagatttttgaaaatttc
tgagatagaaaatcaaatcctagactgtgttaattaaaaaaaacactaatctgagttctgaactgaatgataact
ttcaaattcaagaatacagagttggagaaactatacacaagttaagaaataataactgtcaattagatgtgtctt
cctatccctctagtatagcatcgtcacccttaatatgtaatatgagggtagaagtttggaaattagctgattgga
gttcggcagaagttaagagatggactcattaaagcatatttctaaaattctctctaaaaggttacttttagattt
tggtgtttcgaatatacttatttgtatcagttgtatcatttgcatctccctgcatcctagaaaattgttgagttt
gtcatgagttgcaacttctctgtgcaaggctgcacttaacatagctaaaggaaaacttgatcaaacatatatatg
agtgtaaatgctaaagcttcttaggagaagtgctaccgccacttctttagtggatagtggatactgagttgattt
ctcagcctgtatgaattgaaattgtacttaatatactggtatcgctttcaaatgatgtgcaagagtttgaataac
acattttccttaaaaattcagtgtttagctcttttcattggttaatagtaattattttctttccatgcttacaaa
ctgctgcaattagatggccttctctttaatgcttatatatctaattggtccgaatactaatcccctttctgtact
ctcctcttttcag**TTGAAGACGCTCTTGCACATCCTTACCTAACATCGCTCCATGATATCAGTGACGAGCCCATT
TGCATGACTCCTTTTAGCTTCGACTTTGAGCAGCATGCGCTTACAGAGGAACAGATGAAGGAGCTAATTTACAGG
GAGTCGATTGCATTTAATCCTGAATACCAGCGCATGTGA**

B: cDNA sequence of *Solyc12g019460* (*SlMAPK1*) from LAM183.

ATGGATGGTT**C**CGTTCCGCAAACGGATACGATGATGTCGGATGTGGCTGCACCTCCGGCTCAACAA**C**CACCTCCG
CCGTCACAACCGCTGGC**T**GGAATGGATAATATTCCGGCGACGTTAAGCCATGGTGGCAGGTTCATTCAATACAAT
ATTTTTGGTAATATTTTTGAAGTTACTGCTAAGTATAAACCTCCTATAATGCCAATTGGTAAAGGTGCTTATGGA
ATCGTTTGTTCTGCTTTGAATTCGGAGACAAATGAATCTGTAGCAATTAAGAAAATTGCTAATGCTTTTGATAAC
AAGATTGATGCTAAGAGGACTTTGAGAGAGATCAAGCTTCTTCGACATATGGATCATGAAAATATTGTTGCGATC
AGAGATATAATTCCACCACCACAGAGAGAAGCCTTTAACGATGTTTACATTGCGTATGAGCTTATGGATACTGAT
CTCCATCAAATTATTCGCTCGAATCAGGGTTTATCTGAGGAGCACTGCCAGTATTTCTTGTATCAGATCCTCCGT
GGGTTGAAATACATACATTCTGCAAATGTTTTGCACAGAGAC**T**TAAAGCCTAGCAATCTTCTCTTGAATGCCAAC
TGTGATTTGAAGATATGTGATTTTGGGCTAGCTCGTGTCACTTCTGAAACTGACTTTATGACCGAATATGTTGTG
ACAAGATGGTATCGTCCACCTGAGCTGTTGTTGAATTCATCCGACTATACTGCAGCAATTGATGTATGGTCAGTG
GGTTGCATCTTCATGGAGTTGATGGACAGAAAACCCCTCTTCCCTGGCAGAGATCATGTACACCAGCTGCGTCTT
ATTATGGAGTTGATTGGCACTCCTTCAGAGGCTGAAATGGAATTTT**T**AAATGAGAATGCAAAACGCTATATCCGA
CAACTTCCTCTTTACCGTCGACAATCATTTACTGAAAAGTTCCCGCATGTAAACCCAGCTGCTATTGATCTTGTC
GAGAAAATGTTGACATTTGATCCCAGAAGGAGAATAACAGTTGAAGACGCTCTTGCACATCCTTACCTAACATCG
CTCCATGATATCAGTGACGAGCCCATTTGCATGACTCCTTTTAGCTTCGACTTTGAGCAGCATGCGCTTACAGAG
GAACAGATGAAGGAGCTAATTTACAGGGAGTCGATTGCATTTAATCCTGAATACCAGCGCATGTGA

C: cDNA sequence of *Solyc12g019460* (*SlMAPK1*) from *bif.*

ATGGATGGTT**A**CGTTCCGCAAACGGATACGATGATGTCGGATGTGGCTGCACCTCCGGCTCAACAA**G**CACCTCCG
CCGTCACAACCGCTGGC**C**GGAATGGATAATATTCCGGCGACGTTAAGCCATGGTGGCAGGTTCATTCAATACAAT
ATTTTTGGTAATATTTTTGAAGTTACTGCTAAGTATAAACCTCCTATAATGCCAATTGGTAAAGGTGCTTATGGA
ATCGTTTGTTCTGCTTTGAATTCGGAGACAAATGAATCTGTAGCAATTAAGAAAATTGCTAATGCTTTTGATAAC
AAGATTGATGCTAAGAGGACTTTGAGAGAGATCAAGCTTCTTCGACATATGGATCATGAAAATATTGTTGCGATC
AGAGATATAATTCCACCACCACAGAGAGAAGCCTTTAACGATGTTTACATTGCGTATGAGCTTATGGATACTGAT
CTCCATCAAATTATTCGCTCGAATCAGGGTTTATCTGAGGAGCACTGCCAGTATTTCTTGTATCAGATCCTCCGT
GGGTTGAAATACATACATTCTGCAAATGTTTTGCACAGAGAC**C**TAAAGCCTAGCAATCTTCTCTTGAATGCCAAC
TGTGATTTGAAGATATGTGATTTTGGGCTAGCTCGTGTCACTTCTGAAACTGACTTTATGACCGAATATGTTGTG
ACAAGATGGTATCGTCCACCTGAGCTGTTGTTGAATTCATCCGACTATACTGCAGCAATTGATGTATGGTCAGTG
GGTTGCATCTTCATGGAGTTGATGGACAGAAAACCCCTCTTCCCTGGCAGAGATCATGTACACCAGCTGCGTCTT
ATTATGGAGTTGATTGGCACTCCTTCAGAGGCTGAAATGGAATTTT**A**AAATGAGAATGCAAAACGCTATATCCGA
CAACTTCCTCTTTACCGTCGACAATCATTTACTGAAAAGTTCCCGCATGTAAACCCAGCTGCTATTGATCTTGTC
GAGAAAATGTTGACATTTGATCCCAGAAGGAGAATAACAGTTGAAGACGCTCTTGCACATCCTTACCTAACATCG
CTCCATGATATCAGTGACGAGCCCATTTGCATGACTCCTTTTAGCTTCGACTTTGAGCAGCATGCGCTTACAGAG
GAACAGATGAAGGAGCTAATTTACAGGGAGTCGATTGCATTTAATCCTGAATACCAGCGCATGTGA

D: Protein alignment for *Solyc12g019460* (*SlMAPK1*) comparing LAM183 and *bif* alleles.

```
LAM183    1 MDGSVPQTDTMMSDVAAPPAQQPPPPSQPLAGMDNIPATLSHGGRFIQYNIFGNIFEVTA
bif       1 MDGYVPQTDTMMSDVAAPPAQQAPPPSQPLAGMDNIPATLSHGGRFIQYNIFGNIFEVTA

LAM183   61 KYKPPIMPIGKGAYGIVCSALNSETNESVAIKKIANAFDNKIDAKRTLREIKLLRHMDHE
bif      61 KYKPPIMPIGKGAYGIVCSALNSETNESVAIKKIANAFDNKIDAKRTLREIKLLRHMDHE

LAM183  121 NIVAIRDIIPPPQREAFNDVYIAYELMDTDLHQIIRSNQGLSEEHCQYFLYQILRGLKYI
bif     121 NIVAIRDIIPPPQREAFNDVYIAYELMDTDLHQIIRSNQGLSEEHCQYFLYQILRGLKYI

LAM183  181 HSANVLHRDLKPSNLLLNANCDLKICDFGLARVTSETDFMTEYVVTRWYRPPELLLNSSD
bif     181 HSANVLHRDLKPSNLLLNANCDLKICDFGLARVTSETDFMTEYVVTRWYRPPELLLNSSD

LAM183  241 YTAAIDVWSVGCIFMELMDRKPLFPGRDHVHQLRLIMELIGTPSEAEMEFLNENAKRYIR
bif     241 YTAAIDVWSVGCIFMELMDRKPLFPGRDHVHQLRLIMELIGTPSEAEMEF*---------

LAM183  301 QLPLYRRQSFTEKFPHVNPAAIDLVEKMLTFDPRRRITVEDALAHPYLTSLHDISDEPIC
bif         ------------------------------------------------------------

LAM183  361 MTPFSFDFEQHALTEEQMKELIYRESIAFNPEYQRM
bif         ------------------------------------
```

E: Alignment of the *Solyc12g019460* (*SlMAPK1*) Heinz 1706/LAM183 protein sequence with the closest homologues in *Solanum lycopersicum* and *Arabidopsis thaliana.*

```
SlMAPK1    1 MDG--SVPQTDTMMSDVAAPPAQQPPPPSQPLAGMDNIPATLSHGGRFIQYNIFGNIFEV
SlMAPK2    1 MDG--SAPQTDTVMSDAAAG--QQPAMPPLPMAGMENIPATLSHGGRFIQYNIFGNIFEV
AtMAPK6    1 MDGGSGQPAADTEMTEAPGG--FPAAAPSPQMPGIENIPATLSHGGRFIQYNIFGNIFEV

SlMAPK1   59 TAKYKPPIMPIGKGAYGIVCSALNSETNESVAIKKIANAFDNKIDAKRTLREIKLLRHMD
SlMAPK2   57 TAKYKPPIMPIGKGAYGIVCSALNSETNEHVAIKKIANAFDNKIDAKRTLREIKLLRHMD
AtMAPK6   59 TAKYKPPIMPIGKGAYGIVCSAMNSETNESVAIKKIANAFDNKIDAKRTLREIKLLRHMD

SlMAPK1  119 HENIVAIRDIIPPPQREAFNDVYIAYELMDTDLHQIIRSNQGLSEEHCQYFLYQILRGLK
SlMAPK2  117 HENIVAIRDIIPPPQREAFNDVYIAYELMDTDLHQIIRSNQGLSEEHCQYFLYQILRGLK
AtMAPK6  119 HENIVAIRDIIPPPLRNAFNDVYIAYELMDTDLHQIIRSNQALSEEHCQYFLYQILRGLK

SlMAPK1  179 YIHSANVLHRDLKPSNLLLNANCDLKICDFGLARVTSETDFMTEYVVTRWYRPPELLLNS
SlMAPK2  177 YIHSANVLHRDLKPSNLLLNANCDLKICDFGLARVTSETDFMTEYVVTRWYRPPELLLNS
AtMAPK6  179 YIHSANVLHRDLKPSNLLLNANCDLKICDFGLARVTSESDFMTEYVVTRWYRAPELLLNS

SlMAPK1  239 SDYTAAIDVWSVGCIFMELMDRKPLFPGRDHVHQLRLIMELIGTPSEAEMEFLNENAKRY
SlMAPK2  237 SDYTAAIDVWSVGCIFMELMDRKPLFPGRDHVHQLRLLMELIGTPSEAEMEFLNENAKRY
AtMAPK6  239 SDYTAAIDVWSVGCIFMELMDRKPLFPGRDHVHQLRLLMELIGTPSEEELEFLNENAKRY

SlMAPK1  299 IRQLPLYRRQSFTEKFPHVNPAAIDLVEKMLTFDPRRRITVEDALAHPYLTSLHDISDEP
SlMAPK2  297 IRQLPLYRRQSFVEKFPHVNPAAIDLVEKMLTFDPRRRLTVEDALAHPYLTSLHDISDEP
AtMAPK6  299 IRQLPPYPRQSITDKFPTVHPLAIDLIEKMLTFDPRRRITVLDALAHPYLNSLHDISDEP

SlMAPK1  359 ICMTPFSFDFEQHALTEEQMKELIYRESIAFNPEYQRM
SlMAPK2  357 VCMTPFSFDFEQHALTEEQMKELIYREGLAFNPEYQHM
AtMAPK6  359 ECTIPFNFDFENHALSEEQMKELIYREALAFNPEYQQ-
```

Fig. S6. (A-E). Genomic, cDNA and protein sequences of *SlMAPK1* alleles. Sequences are shown spanning the coding region of *SlMAPK1* (locus name = *Solyc12g019460*). In A, the Heinz 1706 genomic sequence is shown; exon sequences are marked with upper case letters and intron sequences with lower case letters. In B and C the cDNA coding regions are shown for LAM183 and *bif*, respectively. The positions where polymorphisms occur between the LAM183 and *bif* coding regions are highlighted; green, synonymous substitutions; yellow, non-synonymous substitutions; red, conversion of a leucine codon to a stop codon. D shows a protein alignment for LAM183 and *bif*, and stars are colour coded as above to indicate the type of polymorphism; the putative protein kinase activation loop (A-loop) domain is marked with a blue dotted arrow. In E, the protein alignment is

shown between SlMAPK1 and the most closely related tomato (SlMAPK2; *Solyc08g014420*) and Arabidopsis (AtMAPK6; *At2g43790*) proteins. The alignment was created with the boxshade multiple alignment tool (http://www.ch.embnet.org/software/BOX_form.html).

Fig. S7. SNP similarity map for chromosome 12 (87 accessions). An introgression browser (Aflitos *et al.*, 2015) was used to create a SNP similarity map of *bif*, LAM183, Heinz 1706 (labelled as "ref") and 84 resequenced genomes (Aflitos *et. al*. 2014) across chromosome 12. In addition, *S. galapagense* LA0528 data from Lin *et al.* (2014) was included. Bins are set at 50 kb. The grey scale represents the similarity to *bif*, so that a larger number of SNPs results in a darker tone. The red box highlights *bif* and the four accessions of *Solanum galapagense* (LA1044, LA1401, LA0483 and LA0528). The colour scale and top data row represents the total SNP density across all lines. This figure is provided as a separate file to maintain resolution (Supplementary Fig S7.tif).

Fig. S8. SNP similarity map for chromosome 12 (selected accessions). Data is the same as Fig. S7, except that 23 accessions of wild species (*S. habrochaites, S. arcanum, S. pennellii, S. chilense, S. peruvianum, S. huaylasense, S. corneliomulleri, S. chmielewskii and S. neorickii*) were removed from the analysis to increase the contrast within the remaining lines that were more similar to *bif*. The same selected accessions are also used for the similarity tree (Fig. 5, main manuscript). The red box highlights *bif* and the four accessions of *S. galapagense*. This figure is provided as a separate file to maintain resolution (Supplementary Fig S8.tif).

Fig. S9. SNP similarity map for the *BIF* mapping interval (87 accessions). Data is the same as Fig. S7, except that the analysis is restricted to the mapping interval of the *BIF* locus, and the bins are set at 10 kb to increase resolution. The black box highlights the similarity between *bif* and the other *Solanum galapagense* (LA1044, LA1401, LA0483 and LA0528) accessions. Note that a similar analysis is provided as Fig. 6 in the main manuscript, but with fewer lines for brevity. This figure is provided as a separate file to maintain resolution (Supplementary Fig S9.tif).

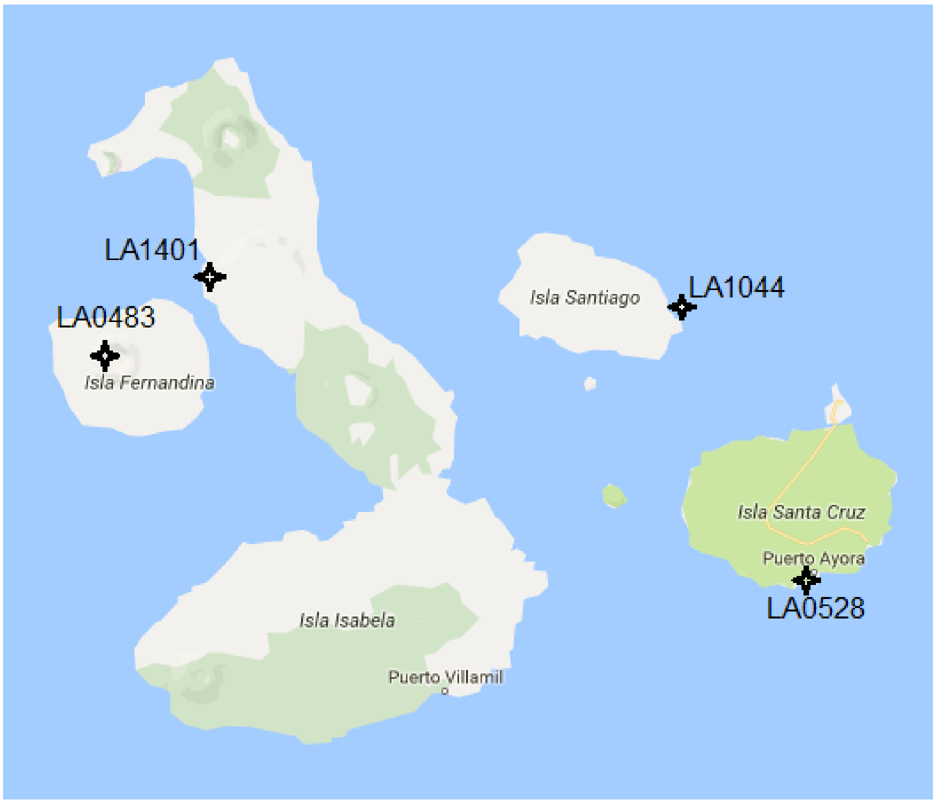

Fig. S10. Collection sites for *Solanum galapagense* accessions. All resequenced accessions of *S. galapagense* known to contain the *bif* allele of *SlMAPK1* are displayed using Google maps and the latitude/longitude co-ordinates from the database of the Tomato Genetics Resource Centre: http://tgrc.ucdavis.edu/.

References referred to in supplementary information

Aflitos S, Schijlen E, Jong H, Ridder D, Smit S, Finkers R, Wang J, Zhang G, Li N, Mao L et al. 2014. Exploring genetic variation in the tomato (*Solanum* section Lycopersicon) clade by whole genome sequencing. *Plant Journal* **80**, 136–148.

Aflitos SA, Sanchez Perez G, Ridder D, Fransz P, Schranz ME, Jong H, Peters SA. 2015. Introgression browser: high-throughput whole genome SNP visualization. *Plant Journal* **82**, 174–182.

Lin T, Zhu G, Zhang J, Xu X, Yu Q, Zheng Z, Zhang Z, Lun Y, Li S, Wang X et al. 2014. Genomic analyses provide insights into the history of tomato breeding. *Nature Genetics* **46**, 1220–1226.
