# Supplementary figures and images for: BIFURCATE FLOWER TRUSS: a novel locus controlling inflorescence branching in tomato contains a defective MAP kinase gene
Source: J Exp Bot. 2018 Mar 2;69(10):2581–93. doi: 10.1093/jxb/ery076 (PMC5920302; doi:10.1093/jxb/ery076)

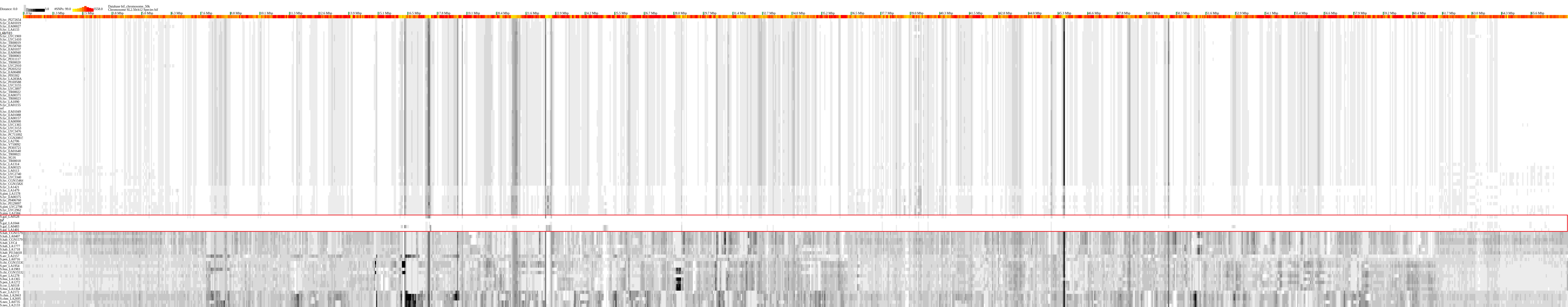

Supplement: Supplementary Figure S7 [file ery076_suppl_supplementary_figure_s7.png]

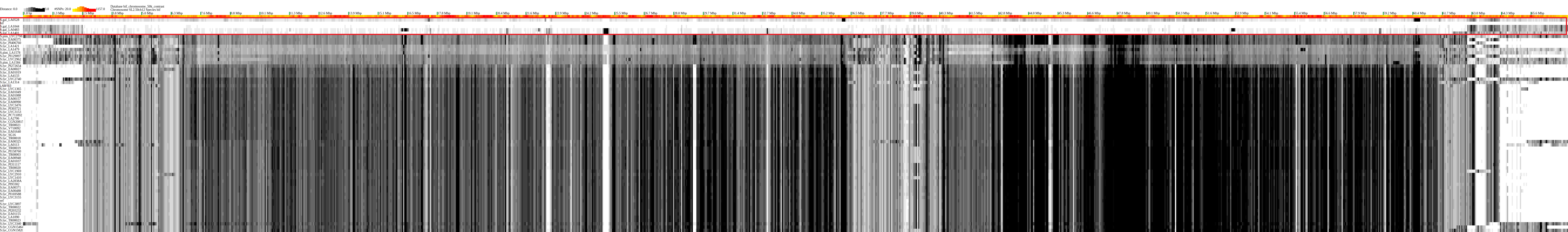

Supplement: Supplementary Figure S8 [file ery076_suppl_supplementary_figure_s8.png]

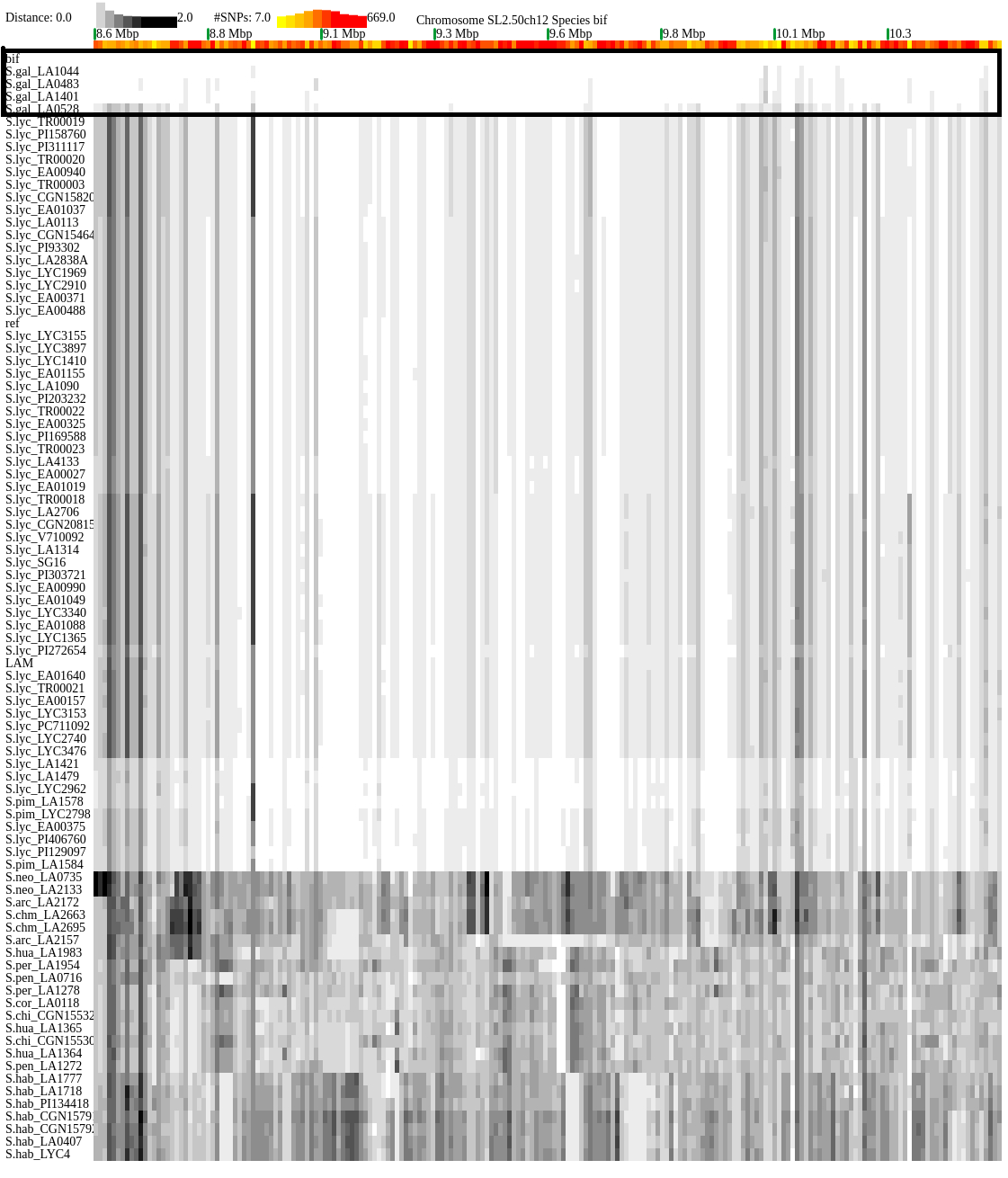

Supplement: Supplementary Figure S9 [file ery076_suppl_supplementary_figure_s9.png]
